# GPER1 as a therapeutic target in MASLD: evidence for steatosis attenuation by agonist G1 in preclinical models

**DOI:** 10.3389/fphar.2026.1764287

**Published:** 2026-03-18

**Authors:** Yifang Li, Jian Jiao

**Affiliations:** Department of Gastroenterology and Hepatology, China-Japan Union Hospital, Jilin University, Changchun, China

**Keywords:** G protein-coupled estrogen receptor 1 (GPER1), GPER1 antagonist (G15), GPER1-specific agonist (G1), hepatic steatosis, metabolic dysfunction-associated steatotic liver disease (MASLD)

## Abstract

**Background:**

Metabolic dysfunction-associated steatotic liver disease (MASLD) is a major cause of liver-related morbidity and mortality. Premenopausal women have a lower MASLD risk than postmenopausal women. G protein-coupled estrogen receptor 1 (GPER1) exerts hepatic protective effects, and GPER1 specific agonist (G1) has shown preclinical potential in improving metabolic disorders. However, clinical studies on G1’s metabolic benefits and GPER1’s clinical relevance in human liver tissue remain unclear. This study aims to bridge basic and clinical research by validating G1’s efficacy in ameliorating MASLD-related hepatic steatosis, exploring its molecular mechanisms, and clarifying GPER1’s association with human MASLD.

**Methods:**

We investigated the expression of GPER1 in human liver tissue and its correlation with the severity of steatosis. The function of GPER1 was validated both *in vitro* (using a free fatty acid-induced hepatocyte steatosis model treated with the GPER1 agonist G1 or antagonist G15) and *in vivo*, with assessments of lipid metabolism-related genes, reactive oxygen species, and apoptosis. GPER1-associated proteins were identified through proteomic sequencing and co-immunoprecipitation.

**Results:**

GPER1 is lowly expressed in MASLD patients, negatively correlating with steatosis severity. G1 upregulates GPER1, alleviates hepatocyte steatosis and lipid deposition, modulates lipid metabolism-related proteins, ameliorates hepatic steatosis, and interacts with GAIP-interacting protein C-terminal 1 (GIPC1). G15 antagonizes these beneficial effects.

**Conclusion:**

Based on clinical data, this study shows that low GPER1 expression correlates closely with hepatic steatosis in MASLD. The GPER1 agonist G1 ameliorates hepatic steatosis via multiple GPER1-dependent mechanisms, including regulating lipid metabolism, suppressing oxidative stress, and reducing apoptosis. Notably, GIPC1 may be involved in the GPER1-mediated regulatory pathway, and its role in this context merits further investigation.

## Graphical Abstract

**Figure:**
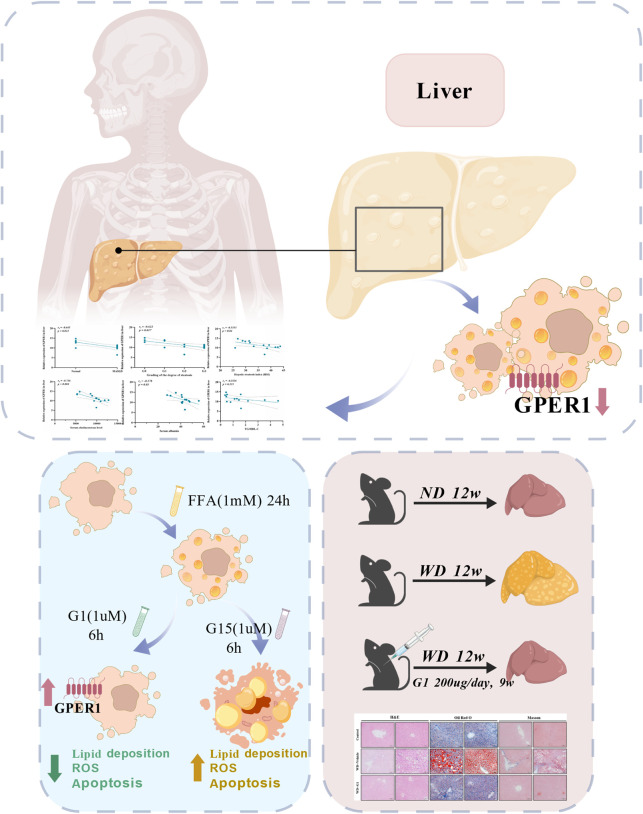
Created with BioGDP.com; (Jiang et al., 2025).

## 1 Introduction

Metabolic dysfunction-associated steatotic liver disease (MASLD) is a pathological syndrome characterized by the excessive deposition of fat in liver cells. It is defined by the presence of steatosis in at least 5% of hepatocytes, along with at least one feature of insulin resistance, in the absence of other causes of secondary hepatic fat accumulation (e.g., excessive alcohol consumption). The global prevalence of MASLD has risen from 25% in 1990–2006 to 38% in 2016–2019, and its incidence is projected to increase by up to 56% by 2030 worldwide (Estes et al., 2018). Furthermore, MASLD is a sex-dimorphic disease with a higher overall prevalence in men. Sex differences in the liver can be influenced by hormonal status throughout life, particularly by circulating estrogen and androgens levels, as well as their ratio (Cherubini et al., 2024). The prevalence of MASLD increases rapidly with advancing age in postmenopausal women. Estrogen replacement therapy (HRT) is used to correct transaminase levels in these women (McKenzie et al., 2003). However, long-term use of estrogen HRT may increase the risk of endometrial cancer, breast cancer, dementia, and stroke in menopausal women (Della Torre, 2026; Dobs et al., 2002). Therefore, it is particularly important to search for drug targets that can replace estrogen to improve liver function in postmenopausal women.

The G protein-coupled estrogen receptor 1 (GPER1), identified 2 decades ago as a novel estrogen receptor, distinct from classical nuclear estrogen receptors (ERs). It primarily mediates rapid, non-genomic signaling, offering a unique pathway for metabolic regulation (Prossnitz and Arterburn, 2015). This mechanistic distinction is crucial, as selective GPER1 agonists such as G1 enable targeted activation without engaging ER pathways, thereby providing a strategy to circumvent the reproductive and feminizing side effects associated with conventional estrogen therapy (DeLeon et al., 2020). Critically, GPER1 activation has been directly implicated in counteracting MASLD. Hepatocyte-specific GPER1 deletion exacerbates hepatic steatosis, inflammation, and insulin resistance in preclinical models (Li et al., 2024). Conversely, GPER1 activation targets key metabolic hubs like AMP-activated protein kinase (AMPK), improving nonalcoholic steatohepatitis (NASH) (Li et al., 2024). This is exemplified by puerarin, a phytoestrogen, which activates the GPER1-AMPK axis to suppress lipogenic genes (FASN, SREBP-1C) and promote lipolysis (ATGL), thereby reducing lipid accumulation (Pham et al., 2022). Moreover, GPER1 knockout mice exhibit metabolic phenotypes without the severe reproductive defects seen in ER knockouts, suggesting a potentially safer therapeutic profile (Filardo and Thomas, 2012). Importantly, the therapeutic potential of targeting GPER1 with specific agonists is well-documented. In diet-induced obese mice, chronic treatment with the selective agonist G1 reduced body weight, adiposity, and circulating lipids by enhancing energy expenditure, mimicking beneficial metabolic effects of estradiol but without inducing uterine growth or other feminizing activities (Kim et al., 2014; Sharma et al., 2020), These findings underscore GPER1’s pivotal role in lipid metabolism and its promise as a novel, safer therapeutic target for fatty liver diseases.

Although GPER1 is known to be important for liver metabolism, there is an unmet need for direct pharmacological evidence of its benefit in MASLD and for a systematic understanding of its downstream effects. Our work attempts to complement existing knowledge by providing an integrated assessment from clinical association to preclinical efficacy using the specific agonist G1 and antagonist G15, and employing an untargeted proteomic approach to analyze the downstream molecular landscape of GPER1 activation. Starting with clinical observations, we integrate evidence from human histology, *in vitro* cytology, and *in vivo* animal studies to demonstrate the beneficial effects of targeting GPER1. This work offers initial proof-of-concept and contribute to the scientific basis for future therapeutic development aimed at this receptor.

## 2 Materials and methods

### 2.1 Materials

Dulbecco’s modified Eagle’s medium (DMEM), penicillin streptomycin solution, and fetal bovine serum (FBS) were purchased from Zhong Qiao Xin Zhou Biotechnology (Shanghai, China). Fatty acid-free bovine serum albumin (BSA) (#A8850) and Oil Red O dye (#G1262) were purchased from Solarbio Science & Technology (Beijing, China). Sodium Oleate (#O7501) and sodium Palmitate (#P9767) were obtained from Sigma-Aldrich (St. Louis, MO, USA). The GPER1-specific agonist (G1, #HY-107216) and the antagonist (G15, #HY-103449) were purchased from MCE (Shanghai, China). The commercial kits for TG (#A110-1-1), TC (#A111-1-1), HDL-C (#A112-1-1) and LDL-C (#A113-1-1) were obtained from Nanjing Jiancheng Bioengineering Institute (Nanjing, China). Reverse transcription kit (#A0010CGQ) and SYBR green qPCR master mix (#A0012-R2) were purchased from EZBioscience (Suzhou, China). For antibody suppliers, please refer to the Supplementary Table S1.

### 2.2 Human liver samples

Human liver samples were obtained from patients who underwent partial hepatectomy for benign liver-occupying lesions at the Biological Sample Bank of the China-Japan Union Hospital of Jilin University. The following factors were excluded in the diagnosis of MASLD using objective imaging, blood tests, and electronic medical records: a history of alcohol consumption and other etiologies of fatty liver disease (e.g., viral hepatitis, drug-induced liver injury, hepatolenticular degeneration, and malnutrition). Each patient was also confirmed to have at least one key component of metabolic syndrome: overweight/obesity, elevated blood pressure/hypertension, prediabetes or type 2 diabetes mellitus, increased triglyceride (TG) levels, and decreased HDL-C levels (Rinella et al., 2023). A total of fourteen patients (seven males and seven females) were included in the study. Human liver samples were categorized into normal and MASLD groups based on the percentage of steatotic hepatocytes relative to the total number of hepatocytes, as determined by hematoxylin and eosin (H&E) staining. Professional pathologists classified liver H&E-stained pathological sections into four grades according to the severity of steatosis in liver tissues: no steatotic hepatocytes were detected in the field of view under the light microscope of H&E staining as grade 0 (G0), steatotic hepatocytes accounted for less than 5% of the total number of hepatocytes as grade 1 (G1), 5%∼33% as grade 2 (G2), and ≥34% as grade 3 (G3). In the normal group, either no steatotic hepatocytes were observed, or the percentage of steatotic hepatocytes accounted for less than 5% of the total hepatocytes within the field of view of H&E-stained sections under a light microscope. Accordingly, six cases with hepatic steatosis grades G0 or G1 were classified as normal, while eight cases with grades G2 or G3 were assigned to the MASLD group. All procedures involving human samples were approved by the Ethics Committee of China-Japan Union Hospital of Jilin University (No. LYSD-2025031202) and adhered to the principles of the Declaration of Helsinki. Informed consent was signed by all participants or their families.

Hepatic Steatosis Index (HSI) is calculated based on the following specific formula = 8×alanine aminotransferase (ALT, IU/L)/aspartate aminotransferase (AST, IU/L) + body mass index (BMI, kg/m^2^) + 2 (if female) + 2 (if type 2 diabetes) (Lee et al., 2010).

### 2.3 Animals and treatment

This study was approved by the Animal Ethics Committee of the School of Public Health, Jilin University (Permit Number: SY:2024-08-005). All animals received humane care, and all experimental protocols were performed in accordance with the Guide for the Care and Use of Laboratory Animals (National Institutes of Health, NIH) and complied with the Animal Welfare Act Regulations. A total of 20 six-week-old female C57BL/6J mice were housed under controlled conditions: constant temperature (20 °C–25 °C), a 12 h light/12 h dark cycle, and ad libitum access to either a normal diet (NC; 4% fat, 18% protein; Cat. No. 1022, Research Diets; supplied by HFKBIO, Beijing, China) or a Western diet (WD; 41% fat, 43% carbohydrate, 17% protein; Cat. No. H10141, Research Diets; supplied by HFKBIO, Beijing, China). Mice were fed these diets for 12 weeks to induce hepatic steatosis and divided into three groups (control group, FFA group, and FFA + G1 group), with six mice randomly assigned to each group. Body weight was measured weekly throughout the experimental period. Prior to sample collection, mice were fasted for 12 h, anesthetize mice by administering 10 mg/mL isopentobarbital via intraperitoneal injection at a dose of 50 mg/kg body weight. Euthanize mice by cervical dislocation after anesthesia. For subcutaneous injections, G1 stock solution was prepared in dimethyl sulfoxide (DMSO, #D8370) and then diluted in the designated vehicle. Mice were subcutaneously injected with either vehicle (5% DMSO, 40% polyethylene glycol 300, 5% Tween 80 in 0.9% sodium chloride) or G1 (200 μg/day), three times per week (Monday, Wednesday, Friday) for 9 weeks (Sharma et al., 2020).

### 2.4 Cell culture and treatment

The HepG2 and Huh7 cell lines were kindly provided by Procell Life Science & Technology Co., Ltd. (Wuhan, China). Cell identification can be found in the supplementary materials. These cells were cultured in complete DMEM, supplemented with 10% FBS and 1% penicillin/streptomycin, and incubated in an atmosphere of 5% CO_2_ at 37 °C. To establish an *in vitro* hepatic steatosis model, HepG2 and Huh7 cells were treated with 1 mM FFA (sodium oleate: sodium palmitate at a 2:1 M ratio) in a complete medium containing 1% fatty acid-free BSA for 24 h. Control cells received complete medium containing only 1% fatty acid-free BSA. After induction, cells were washed twice with PBS and subsequently treated with either G1 (1 μM) or G15 (1 μM) for 6 h in both cell lines. Biochemical assays were performed following the treatment.

### 2.5 Oil Red O staining

HepG2 and Huh7 cells were seeded into 6-well plates at a density of 1 × 10^5^ cells per well. For intracellular Oil Red O staining, the working solution was prepared by diluting the Oil Red O stock solution with distilled water at a 3:2 ratio, the mixture was then allowed to settle at room temperature for 20 min. Subsequently, the fixed cells were incubated with this Oil Red O working solution for 30 min to complete the staining process.

### 2.6 Determination of intracellular, tissue lipid content and serum biochemistry

Lipid levels were quantified in cell lysates, liver tissue homogenates, and serum samples. For intracellular measurements, HepG2 and Huh7 cells were washed, sonicated in PBS, and the lysates were assayed for triglyceride (TG) and total cholesterol (TC) using commercial kits (#A110-1-1 and #A111-1-1) obtained from Nanjing Jiancheng Bioengineering Institute (Nanjing, China). Results were normalized to total protein concentration determined by a BCA assay. Serum and hepatic tissue lipid levels (TG, TC, HDL-C, LDL-C) were measured using a Chemray 800 automatic biochemical analyzer (Rayto, Shenzhen, China). All procedures followed the manufacturers’ instructions.

### 2.7 Flow cytometry detection of cell apoptosis

The Annexin V-FITC/PI dual staining method combined with flow cytometry was employed to quantitatively analyze apoptosis rates. The Annexin V-FITC/PI Apoptosis Detection Kit (#A5001-02P-L) was purchased from Tianjin Simu Biotech Co., Ltd. Samples were prepared according to manufacturer guidelines, with all procedures completed within 1 h. Gates were set on the forward scatter (FSC-A) vs. side scatter (SSC-A) scatter plot to select morphologically intact single-cell populations. Cell debris (low FSC, low SSC) and cell aggregates (high FSC, high SSC) were excluded to ensure homogeneity of the analyzed population (Supplementary Figures S7–S9). In the FITC (Annexin V) vs. PI two-parameter scatter plot, establish quadrant gates based on unstained and single-stained controls to clearly distinguish cell populations with different staining characteristics, preventing misinterpretation of results due to fluorescence spillover. Data were collected using a flow cytometer (Becton CYTOMINCS FC500, USA) and analyzed with FlowJo v10.8.1 software.

### 2.8 Flow cytometry detection of intracellular reactive oxygen species (ROS) levels

Quantitative detection of total intracellular ROS levels was achieved using the DCFH-DA fluorescent probe in combination with flow cytometry. Sample preparation and staining were performed according to the manufacturer’s instructions. A two-parameter scatter plot was constructed using forward scatter area (FSC-A) versus side scatter area (SSC-A). A polygonal gate was used to select the core cell population. This selection ensures subsequent analysis targets morphologically intact single cells, minimizing interference from non-target components on ROS detection signals. Set a rectangular gate along the diagonal of the single-cell distribution. This gate further eliminates incompletely dispersed cell aggregates, significantly enhancing the reliability of detection data. Gating strategy of the flow cytometric ROS analysis are shown in Supplementary Figures S11, S13. Data were acquired using a flow cytometer (Becton Dickinson FC500, USA) and analyzed with FlowJo v10.8.1 software.

### 2.9 Quantitative real-time RT-qPCR analysis

Total RNA was extracted from cells or liver tissues by Trizol reagent (Thermo). Total RNA (1,000 ng) was subjected to reverse transcription to synthesize cDNA with a color reverse transcription kit following the manufacturer’s protocol. Quantitative real-time qPCR was performed using the SYBR green qPCR master mix on the Realplex Eppendorf real-time PCR detection system. The sequences for the primers used in this study are listed in Supplementary Table 2 β-actin was used as a reference gene for all samples. Relative gene expression was calculated after normalization to β-actin following the 2^−ΔΔCT^ method.

### 2.10 Western blotting analysis

Total protein was extracted from liver tissues or cells using standard protocols, and protein concentrations were determined via a BCA protein assay kit. A total of 40–80 μg of protein was loaded onto 10% sodium dodecyl sulfate-polyacrylamide gel electrophoresis (SDS-PAGE) gels. Membranes were blocked with 5% non-fat dry milk in Tris-buffered saline with Tween 20 (TBST) at room temperature for 1 h, then incubated overnight at 4 °C with primary antibodies. β-actin was used as the loading control. Protein signals were detected using a Dual-Color Infrared Laser Imaging System (Licor Odyssey, Lincoln, NE, USA). The optical density (OD) of target protein bands was quantified using ImageJ software (version 2.14.0).

### 2.11 Co-immunoprecipitation (Co-IP) assay​

Co-IP experiments were conducted with an IP/Co-IP kit purchased from Thermo Pierce (Rockford, IL, USA), following the manufacturer’s recommended protocol. For each assay, cellular lysates containing 500 μg of total protein were aliquoted into microcentrifuge tubes, and 5 μg of the target immunoprecipitation (IP) antibody was added to each tube. These mixtures were then placed on a rotator at 4 °C for overnight incubation to ensure efficient antigen-antibody complex formation.​ On the following day, pre-washed A/G agarose beads were added to each tube containing the antigen-antibody complexes, and the samples were incubated at 37 °C for 1 h to facilitate bead binding. After incubation, the beads were subjected to three sequential washes with ice-cold IP lysis/wash buffer.​ To elute the immunoprecipitated proteins from the beads, 3× SDS-PAGE loading buffer was added to each sample, and the mixtures were heated to 95 °C–100 °C for 5 min. The eluted protein fractions were then collected by centrifugation and immediately analyzed via Western blotting to detect the target proteins.

### 2.12 H&E staining

H&E staining was performed to assess lipid accumulation and inflammatory infiltration in liver tissues. Liver tissues were fixed in 4% neutral buffered formalin and embedded in paraffin. Sections were prepared and processed according to standard H&E protocols. Briefly, paraffin sections were deparaffinized and rehydrated by immersion in xylene substitute for 20 min, followed by a graded ethanol series (100%, 95%, 85%, and 75%) and finally rinsed with distilled water. The sections were then stained with hematoxylin for 5 min, rinsed under running tap water, differentiated briefly in acid alcohol, rinsed again, and blued in warm water before a final tap water rinse. Subsequently, the sections were counterstained with eosin for 10 s. Dehydration was carried out through a reverse graded ethanol series (75%, 85%, 95%, and 100%), followed by clearing in xylene twice. The sections were finally mounted with neutral resin. Images were captured and analyzed using an upright microscope (Olympus, Tokyo, Japan).

### 2.13 Immunohistochemical staining

Liver tissue sections were first blocked with 5% BSA in PBS at room temperature for 1 h, then incubated overnight at 4 °C with the primary antibody: rabbit anti-human GPER1 (ab39742, 1:100 dilution, Abcam, USA) at a 1:100 dilution. The following day, sections were incubated with the secondary antibody—goat anti-rabbit IgG (Cat. No. GB23303, Servicebio, Wuhan, China)—at a 1:200 dilution for 1 h at room temperature. Images were acquired using an upright microscope (Olympus, Tokyo, Japan) and quantified using ImageJ software (version 2.14.0).

### 2.14 Untargeted proteomics analysis

Global proteomic profiling was performed using label-free quantitative proteomics via liquid chromatography-tandem mass spectrometry (LC-MS/MS) (Shanghai iProteome Biotechnological Co., Ltd, Shanghai, China). Briefly, we first prepared cell pellets from three experimental groups (Control, FFA, and FFA + G1) following the aforementioned cell treatment procedure, with three biological replicates per group, resulting in a total of nine samples for proteomic analysis. Protein extraction and concentration determination were subsequently performed. The quality of the mass spectrometry data was assessed by analyzing parameters including peptide ion score distribution, dynamic range of protein quantification, and peptide sequence length distribution. Finally, qualitative and quantitative analyses of the identified proteins were conducted, encompassing overall differential protein analysis and screening of significantly altered proteins. Principal Component Analysis (PCA) (Supplementary Figure S2), Gene Ontology (GO) and Kyoto Encyclopedia of Genes and Genomes (KEGG) enrichment analysis of proteins upregulated by G1 in FFA-induced cells were performed using R software (version 4.5.0; R Development Core Team; http://R-project.org). Detailed procedures are described in the Supplementary Materials.

### 2.15 Statistical analysis

Data are expressed as the mean ± standard error of the mean (SEM) from three independent experiments. Statistical analyses were performed using SPSS 25.0 software, and graphs were generated using GraphPad Prism 9.0 software. For comparisons between two groups, an unpaired Student’s t-test was used. For multiple comparisons in cellular models, one-way analysis of variance (ANOVA) was performed, followed by Dunnett’s post-hoc test to compare each experimental group with the control group. Spearman’s rank correlation analysis was employed to assess the association between two continuous variables. Two-tailed P-values were calculated, and a P-value <0.05 was considered statistically significant.

## 3 Results

### 3.1 GPER1 expression decreases in human liver tissue and negatively correlates with the severity of hepatic steatosis

**FIGURE 1:**
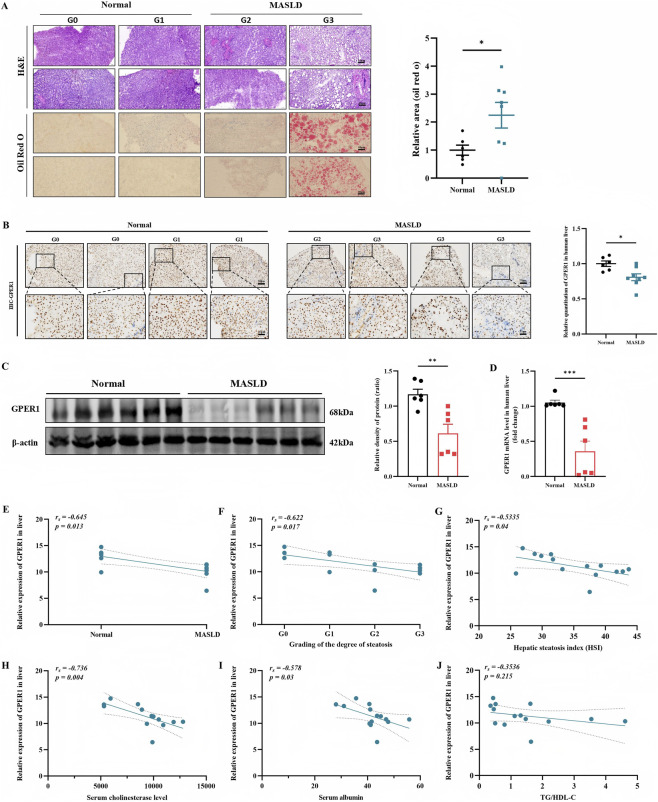
GPER1 expression decreases in human liver tissue and negatively correlates with the severity of hepatic steatosis. **(A)** Representative images of H&E-stained and Oil Red O-stained sections from MASLD and normal liver tissue, with two representative sections selected for each grade. Scale bar, 50 μm; G0 indicates no fatty hepatocytes observed in the H&E-stained light microscopic field; G1 indicates fatty hepatocytes constitute <5% of total hepatocytes; G2 indicates fatty hepatocytes constitute 5%–33% of total hepatocytes; G3 indicates fatty hepatocytes constitute ≥34% of total hepatocytes. Quantitative analysis of Oil Red O staining in MASLD and normal liver tissue sections (n = 6–8). **(B)** Representative IHC images and quantitative analysis of GPER1 expression in liver tissues from individuals with or without MASLD (n = 6–8). Brown deposits indicate positive staining for GPER1, visualized using the 3,3′-diaminobenzidine (DAB) substrate. Nuclei are counterstained with hematoxylin (blue). Scale bar, 25–50 μm. **(C)** Western blot analysis of GPER1 protein expression in liver tissues with or without MASLD (n = 6). The relative protein intensities were normalized to β-actin. **(D)** RT-qPCR detection of GPER1 mRNA levels in liver tissues with or without MASLD (n = 6). **(E)** Correlation analysis between GPER1 protein expression and MASLD presence in human liver samples (n = 14). **(F)** Correlation analysis between GPER1 protein expression and the degree of hepatic steatosis in human liver samples (n = 14). **(G)** Correlation analysis between GPER1 protein expression and the hepatic steatosis index (HSI) in human liver samples (n = 14). **(H–J)** Correlation analysis between GPER1 protein expression and the serum cholinesterase level, serum albumin, and the ratio of TG/HDL-C (n = 14). Data are expressed as mean ± standard error of the mean (Mean ± SEM). *P < 0.05. Student’s unpaired t-test was used for data statistics in Figures **(A–D)**. Spearman’s correlation analysis was used for data statistics in Figures **(E–J)**. ^*^ *P* < 0.05, ^**^ *P* < 0.01, ^***^ *P* < 0.001.

We observed significantly increased lipid droplet deposition in liver tissue from MASLD patients compared to the control group using H&E and Oil Red O staining (Figure 1A). Subsequent immunohistochemistry, Western blot, and RT-qPCR analyses revealed reduced GPER1 expression in human liver tissue (Figures 1B–D). Correlation analysis showed that GPER1 expression levels were negatively associated with MASLD presence (r = −0.645, p = 0.013) (Figure 1E), the severity of steatosis (r = −0.622, p = 0.017) (Figure 1F), and the hepatic steatosis index (r = −0.5335, p = 0.04) (Figure 1G). There were also negative correlations with serum albumin (r = −0.736, p = 0.004) and serum cholinesterase levels (r = −0.578, p = 0.03) (Figures 1H,I). However, there was no significant correlation with TG/HDL-C (r = −0.3536, p = 0.215) (Figure 1J).

### 3.2 G1 upregulates GPER1 protein expression, improves hepatic lipid deposition, reduces reactive oxygen species (ROS) production, and decreases hepatocyte apoptosis *in vitro*

We first established an FFA-induced MASLD hepatocyte model *in vitro* and determined the optimal working concentration of FFA using the CCK-8 assay (Supplementary Figure S1A). Our results showed that 1 mM FFA exerted no cytotoxic effect on HepG2 and Huh7 cells while effectively inducing cellular steatosis. This concentration has been widely used in previous studies (Wang et al., 2022; Ricchi et al., 2009). As expected, Oil Red O staining revealed a significant increase in lipid droplet accumulation in FFA-treated hepatocyte cell lines compared with the control group (Supplementary Figures S1B, C). Furthermore, intracellular triglyceride (TG) content was significantly higher in the FFA-treated group (Supplementary Figure S1D). Additionally, the expression levels of genes related to fatty acid synthesis (FASN,SCD1,ACC1) were upregulated in the FFA group (Supplementary Figure S1E).

**FIGURE 2:**
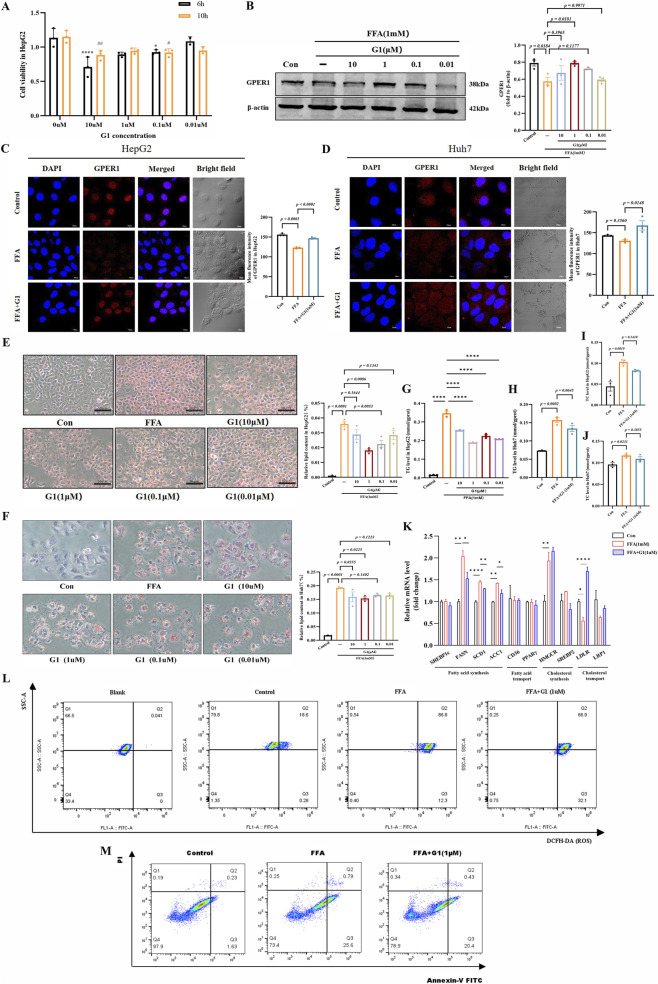
G1 upregulates GPER1 protein expression, improves hepatic lipid deposition, reduces reactive oxygen species (ROS) production, and decreases hepatocyte apoptosis *in vitro*. **(A)** CCK8 assay was performed to assess the viability of HepG2 cells treated with varying concentrations of G1. **(B)** Western blot detection of GPER1 protein expression at different G1 concentrations. The relative intensities of proteins were normalized to β-actin. **(C,D)** Representative immunofluorescence staining images show GPER1 expression in FFA (1 mM)-induced HepG2 and Huh7 cells treated with G1 (1 µM). Quantitative data represent the mean fluorescence intensity ±SEM of multiple fields from three independent experiments. Scale bar, 20 μm. **(E,F)** Representative Oil Red O-stained images and quantitative analysis of the lipid droplet accumulation in HepG2 and Huh7 cells following FFA (1 mM) induction and subsequent treatment with various concentrations of G1. Scale bar, 50 µm. **(G,H)** TG levels in HepG2 and Huh7 cells were measured after FFA (1 mM) induction and treatment with G1. **(I,J)** TC levels in HepG2 and Huh7 cells were measured after FFA (1 mM) induction and treatment with G1 (1uM). **(K)** The relative mRNA expression of genes related to fatty acid synthesis, transport, cholesterol synthesis, and cholesterol transport in HepG2 cells induced by FFA (1 mM) and then treated with G1 (1 µM). **(L)** ROS levels measured by flow cytometry in HepG2 cells. Cells were loaded with the fluorescent probe DCFH-DA. **(M)** Flow cytometry analysis of apoptosis in HepG2 cells. Cells were stained with Annexin V-FITC and PI. Lower left quadrant: viable cells; upper left quadrant: necrotic cells; lower right quadrant: early apoptotic cells; upper right quadrant: late apoptotic cells. Data are presented as the mean ± SEM of three biologically independent experiments (n = 3). Statistical analyses were performed using one-way ANOVA with post hoc multiple comparisons by Dunnett’s method. Compared with the FFA group, ^*^ *P* < 0.05, ^**^ *P* < 0.01, ^***^ *P* < 0.001, ^****^ *P* < 0.0001. Compared to treatment without G1 for 10 h, ^#^ *P* < 0.05, ^##^ *P* < 0.01, ns indicates no statistical significance.

The CCK8 assay revealed that 1 μM G1 exhibited no significant toxic effects on HepG2 cells after 6 h of incubation (Figure 2A). Subsequent experiments were conducted at this concentration. Western blot and immunofluorescence analyses demonstrated decreased GPER1 expression in the FFA-induced steatotic hepatocyte model, whereas G1 (1 μM) significantly upregulated GPER1 protein expression in HepG2 and Huh7 cells (Figures 2B–D). Concurrently, Oil Red O staining revealed that G1 (1 μM) reduced lipid droplet accumulation (Figures 2E,F) and TG levels (Figures 2G,H) in HepG2 and Huh7. While the effect at 10 μM was attenuated, consistent with the observed cytotoxicity at this higher dose. Notably, the TC levels in HepG2 and Huh7 were unaltered by G1 treatment (Figures 2I,J). This differential effect on TG versus TC suggested a selective action on specific metabolic pathways. Consistently, RT-qPCR results showed that G1 decreased mRNA levels of fatty acid synthesis-related genes (FASN, SCD1, ACC1) (Figure 2K), while leaving the mRNA levels of key genes involved in cholesterol synthesis (HMGCR, SREBP2) unaffected (Figure 2K). Flow cytometry data revealed that G1 attenuated FFA-induced ROS production slightly (Figure 2L) and led to a modest reduction in apoptosis (Figure 2M) in HepG2 cells.

### 3.3 G15 downregulates GPER1 protein expression, exacerbates hepatic lipid accumulation, promotes ROS production, and accelerates hepatocyte apoptosis *in vitro*

**FIGURE 3:**
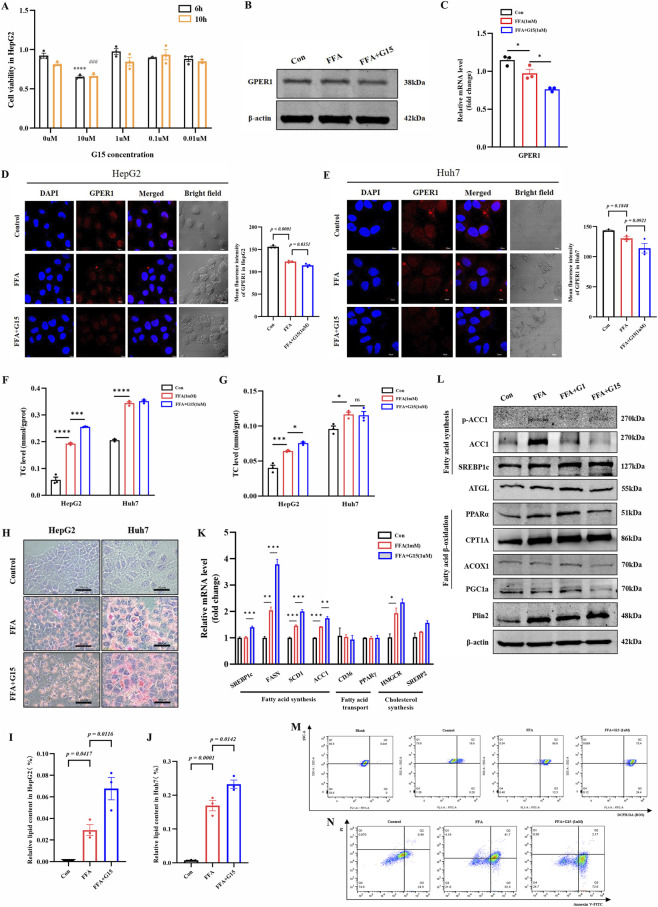
G15 downregulates GPER1 protein expression, exacerbates hepatic lipid accumulation, promotes ROS production, and accelerates hepatocyte apoptosis *in vitro*. **(A)** CCK8 assay was performed to assess the viability of HepG2 cells treated with varying concentrations of G15. **(B,C)** Western blot and RT-qPCR detection of GPER1 after the treatment of G15 (1 µM). **(D,E)** Representative immunofluorescence staining images and quantitative analysis of GPER1 expression in FFA (1 mM)-induced HepG2 and Huh7 cells treated with G15 (1 µM). Quantitative data represent the mean fluorescence intensity ±SEM of multiple fields from three independent experiments. Scale bar, 20 μm. **(F,G)** TG and TC levels in HepG2 and Huh7 cells were measured after FFA (1 mM) induction and treatment with G15 (1uM). **(H–J)** Representative Oil Red O-stained images and quantitative analysis of the lipid droplet accumulation in HepG2 and Huh7 cells following FFA (1 mM) induction and subsequent treatment with various concentrations of G15. Scale bar, 50 µm. **(K)** The relative mRNA expression of genes related to fatty acid synthesis, transport, and cholesterol synthesis in HepG2 cells induced by FFA (1 mM) and then treated with G15 (1 µM). **(L)** Western blot analysis was used to assess the effects of G1 and G15 on fatty acid synthesis and fatty acid oxidation-related protein expression in FFA-induced HepG2 cells. **(M)** ROS levels measured by flow cytometry in HepG2 cells. Cells were loaded with the fluorescent probe DCFH-DA. **(N)** Flow cytometry analysis of apoptosis in HepG2 cells. Cells were stained with Annexin V-FITC and PI. Lower left quadrant: viable cells; upper left quadrant: necrotic cells; lower right quadrant: early apoptotic cells; upper right quadrant: late apoptotic cells. Data are presented as the mean ± SEM of three biologically independent experiments (n = 3). Statistical analyses were performed using one-way ANOVA with post hoc multiple comparisons by Dunnett’s method. Compared with the FFA group, ^*^ *P* < 0.05, ^**^ *P* < 0.01, ^***^ *P* < 0.001, ^****^ *P* < 0.0001. Compared to treatment without G1 for 10 h, ^#^ *P* < 0.05, ^###^ *P* < 0.001, ns indicates no statistical significance.

We found that the CCK8 assay detected no significant toxic effects of 1 μM G15 on HepG2 cells after 6 hours of treatment, confirming its suitability for subsequent experiments (Figure 3A). Western blot, RT-qPCR, and immunofluorescence analyses revealed that G15 downregulates GPER1 protein expression (Figures 3B–E). Additionally, G15 increased TG levels (Figure 3F) and lipid droplet accumulation (Figures 3H–J) in HepG2 and Huh7 cells, with a slight elevation in TC levels (Figure 3G). RT-qPCR showed that G15 increased the mRNA levels of genes related to fatty acid synthesis (SREBP1c, FASN, SCD, and ACC1) (Figure 3K). Western blot analysis revealed that G15 decreased the expression of proteins related to fatty acid oxidation (PPARα, CPT1A, ACOX1, and PGC1α), whereas G1 increased their expression (Figure 3L). G15 showed minimal impact on both ROS levels and apoptosis induced by FFA in HepG2 cells, as determined by flow cytometry (Figures 3M–N).

### 3.4 G1 improves hepatic steatosis and lipid metabolism in mice induced by a western diet

**FIGURE 4:**
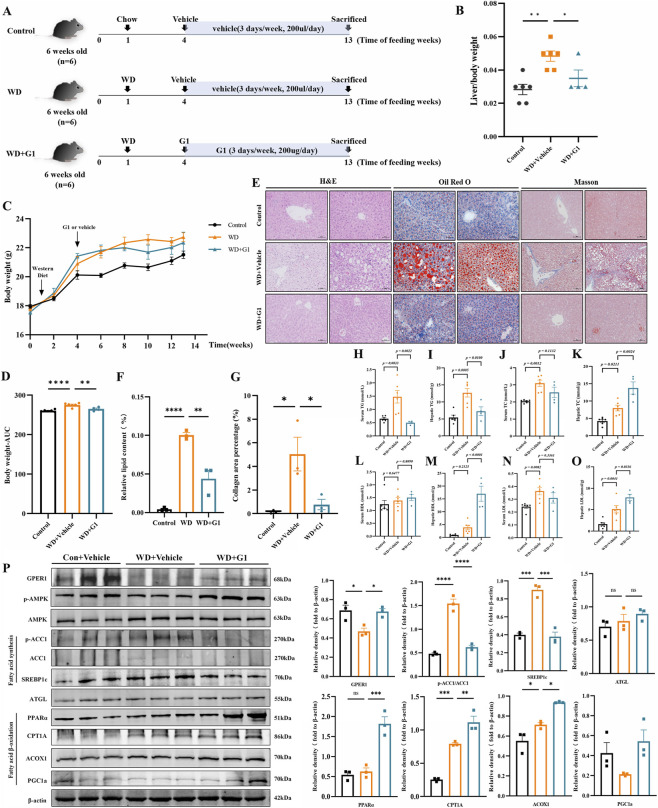
G1 improves hepatic steatosis and lipid metabolism in mice induced by a Western diet. **(A)** Schematic diagram of the groupings for the WD-induced MASLD mouse models. Mice were acclimatized for 1 week prior to being fed a normal diet or WD. Starting from the end of week 4, the G1 group received subcutaneous injections of G1 (200 μg/day, three times per week) for 9 weeks, while the control and WD groups received subcutaneous injections of the G1 solvent. **(B,C)** Changes in liver weight **(B)** and body weight **(C)**. **(D)** Area under curve (AUC) analysis of the body weight. **(E)** Representative images of liver sections stained with H&E staining, Oil Red O staining, and Masson’s trichrome staining. **(F)** Quantification of Oil Red O staining shows the percentage of lipid droplet area per field. **(G)** Quantification of Masson’s trichrome staining shows the percentage of collagen fiber area relative to the total tissue area. Scale bar, 50 μm. **(H)** Serum TG levels. **(I)** Hepatic TG levels. **(J)** Serum TC levels. **(K)** Hepatic TC levels. **(L)** Serum HDL levels. **(M)** Hepatic HDL levels. **(N)** Serum LDL levels. **(O)** Hepatic LDL levels. **(P)** Western blot analysis quantifying the expression of fatty acid synthesis-related proteins (P-ACC1, ACC1, and SREBP1c) and fatty acid oxidation-related proteins (PPARα, PGC1α, CPT1A, and ACOX1) in liver tissues from the control, WD, and WD + G1 groups. The relative intensities of proteins were normalized to β-actin. Statistical analysis was performed using one-way ANOVA followed by Dunnett’s post hoc test was performed (n = 4–6 mice per group). ^*^ *P* < 0.05, ^**^ *P* < 0.01, ^***^ *P* < 0.001, ^****^ *P* < 0.0001 vs. the WD group, ns indicates no statistical significance.

A mouse model of MASLD was induced by feeding mice a WD for 12 weeks, with the control group receiving a standard diet (grouping as shown in Figure 4A). Compared to standard diet mice, WD mice exhibited significantly increased liver weight and body weight (Figures 4B,D). Mice treated with G1 began to show reduced body weight compared to WD mice starting from week 4 (Figure 4C), and ultimately exhibited lower liver weight than WD mice (Figure 4B). Oil Red O staining revealed markedly increased lipid droplets in the livers of WD mice compared to those on a standard diet, while lipid droplet accumulation was significantly reduced in G1-treated mice relative to the WD group (Figures 4E,F). H&E staining revealed numerous lipid vacuoles within hepatocytes and extensive fatty infiltration in the livers of the WD group. In contrast, hepatocytes in the G1-treated group exhibited relatively clear morphology and boundaries, with reduced lipid vacuoles in the cytoplasm (Figure 4E). Masson staining demonstrated increased collagen fibers in the liver tissue of the WD group, which were reduced in the G1-treated group (Figures 4E,G).

Additionally, we assessed lipid metabolism levels in mice. Serum and hepatic triglyceride (TG) levels were significantly increased in WD-induced mice compared to the control group (Figures 4H,I), indicating severe lipid metabolism dysfunction induced by the Western diet. Compared to the WD group, mice treated with G1 exhibited reduced serum TG and hepatic TG levels (Figures 4H,I). Serum TC levels were lower than in the WD group (Figure 4J), while hepatic TC levels were elevated (Figure 4K). mice treated with G1 exhibited significantly higher hepatic HDL and LDL levels than WD mice, while serum HDL and LDL levels were slightly lower than in the WD group (Figures 4L–O). Furthermore, compared with the WD group, mice treated with G1 exhibited decreased expression of fatty acid synthesis-related proteins (pACC1/ACC1, SREBP1c) and increased expression of fatty acid oxidation-related proteins (CPT1A, ACOX1) in the liver (Figure 4P).

### 3.5 G1 upregulates GIPC1 expression and GPER1 interacts with GIPC1

**FIGURE 5:**
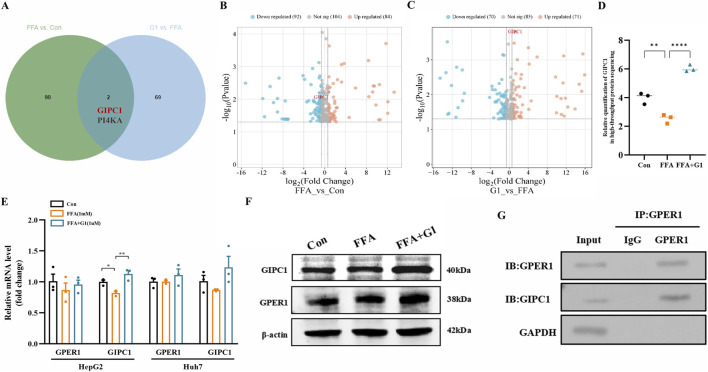
G1 upregulates GIPC1 expression and GPER1 interacts with GIPC1. **(A)** Venn diagram showing the number of genes co-occurring in the expression of downregulated differential genes in the FFA group compared to the control group versus the expression of upregulated differential genes in the FFA + G1 group. **(B,C)** Volcano map showing the differential gene expression in the FFA group compared to the control group (left) and in the FFA + G1 group compared to the FFA group (right). **(D)** The expression of GIPC1 in proteomics sequencing of the control group, FFA group, and FFA + G1 group cells. **(E,F)** The relative mRNA and protein expression of GPER1 and GIPC1 in control, FFA, and FFA + G1 groups of cells was determined by RT-qPCR and Western blot. The relative intensities of proteins were normalized against β-actin. **(G)** Co-immunoprecipitation (Co-IP) assay confirming the interaction between GPER1 and GIPC1 proteins. Data are presented as the mean ± SEM of three biologically independent experiments (n = 3). Figures **(D,E)** were performed using one-way ANOVA with post hoc multiple comparisons by Dunnett’s method. Con, control; FFA, free fatty acid.

We performed untargeted LC-MS/MS-based proteomics in FFA-induced HepG2 cells treated with G1, a specific agonist of GPER1. Using the absolute value of fold change |FC|≥1.5, p < 0.05 as the screening criterion for differential proteins, we took the intersection of 92 differential proteins whose expression was downregulated in the FFA group and 71 differential proteins whose expression was upregulated in the FFA + G1 group (Figure 5A) (Bardou et al., 2014), and found that the expression of GIPC1 was downregulated in HepG2 cells after FFA induction, whereas the expression was upregulated in the G1-treated cells (Figures 5B–D). We further verified this by Western blot and RT-qPCR assays (Figures 5E,F). In the context of the FFA-induced MASLD cell model, activation of GPER1 upregulated GIPC1 expression. To further confirm the relationship between GPER1 and GIPC1, we conducted Co-IP experiments. The results showed that the GPER1 IP antibody successfully pulled down the GIPC1 protein, demonstrating that a certain degree of interaction exists between the two proteins (Figure 5G).

KEGG pathway enrichment analysis of the proteins upregulated by G1 in FFA-induced cells revealed significant enrichment in several fundamental metabolic processes, most notably the biosynthesis of amino acids and the pentose phosphate pathway. Notably, no canonical lipid metabolism pathways were significantly enriched among the G1-upregulated proteins (Supplementary Figure S3). GO biological process enrichment analysis corroborated and extended the findings from KEGG pathway analysis. The most significantly enriched processes included organophosphate metabolic process, notably, the pentose-phosphate shunt, which exhibited an exceptionally high enrichment score. Processes related to nucleotide metabolism and epithelial cell migration were also prominently represented among the G1-upregulated proteins (Supplementary Figure S4). The downregulated proteome in G1-treated cells showed enrichment in nicotinamide and cholesterol metabolism pathways. However, the enrichment significance (FDR >0.05) for these pathways did not reach statistical significance (Supplementary Figures S5, S6).

## 4 Discussion

The emerging role of GPER1 in metabolism presents a promising therapeutic target for metabolic dysfunction-associated steatotic liver disease (MASLD). The identification of the first GPER1 specific agonist G1 was described in 2006 (Bologa et al., 2006), and by 2020, its therapeutic potential in improving conditions such as obesity and diabetes in preclinical models was established, through mechanisms like enhancing insulin sensitivity and regulating metabolism (Liu et al., 2025). Notably, among known GPER1 targeting ligands, the G1 isomer LNS8801 has advanced to clinical trials for cancer therapy (Natale et al., 2025), underscoring the pharmacological tractability of this target, though its metabolic benefits await clinical investigation in MASLD. Recent mechanistic studies, including work by Li and colleagues in early 2024, have elucidated that GPER1 activation improves NASH through AMPK in mice (Li et al., 2024). Other investigations, such as phytoestrogen have shown GPER1 activation can suppress hepatic lipogenesis (Farruggio et al., 2020). However, these valuable studies have primarily employed genetic or compound tools at the cellular or animal level, leaving a gap in directly linking GPER1 to human pathology. This study expands this by building a translational bridge from clinical observation to mechanistic validation. We first analyzed the association between GPER1 expression and steatosis severity in human liver samples. And then provided pharmacological proof-of-concept using the specific agonist G1 and antagonist G15, demonstrating that specific GPER1 activation is sufficient to attenuate steatosis in preclinical models. Furthermore, the untargeted proteomic analysis extends the mechanistic understanding beyond established pathways. We reveal that G1 orchestrates a broader metabolic reprogramming, including the upregulation of the pentose phosphate pathway, and identifies novel potential effectors (GIPC1). Collectively, the findings not only validate GPER1 as a druggable target using specific pharmacological agents but also expand the mechanistic landscape, refining the conceptual framework for targeting GPER1 in MASLD.

The HSI index serves as a simple and efficient screening tool for MASLD (Lee et al., 2010; Song et al., 2022; Yao et al., 2023), and we employed it as a positive control. Analysis of human liver samples showed that GPER1 exhibits low expression in MASLD, with its expression levels negatively correlated with the degree of hepatic steatosis (both in our sample grading and HSI index calculations). This suggests that high GPER1 expression may act as a protective factor in MASLD. Although the small sample size may limit statistical power, to our knowledge, this represents the first multi-method validation of GPER1 expression in MASLD liver tissue. Therefore, we used G1 to activate GPER1, thereby confirming its ability to increase GPER1 protein expression and reduce FFA-induced hepatic steatosis and lipid accumulation. At the same time, G1 reduced the mRNA levels of genes related to fatty acid synthesis while moderately increasing the levels of proteins associated with fatty acid oxidation, which is consistent with prior studies (Pham et al., 2022). However, the data demonstrate that G1 may exert a selective effect on hepatic lipid metabolism in HepG2. Specifically, G1 potently inhibited *de novo* lipogenesis, as shown by the downregulation of FASN, ACC1, and SCD1, leading to a significant reduction in TG accumulation and lipid droplets. Crucially, this effect was not due to a generalized suppression of all lipid pathways. The absence of change in total cholesterol levels, coupled with unaltered expression of cholesterol synthesis genes (HMGCR, SREBP2). This specificity strengthens the potential of G1 as a targeted modulator. It is important to note that the dose-response relationship of G1 *in vitro* model was non-monotonic. The superior efficacy of 1 μM over 10 μM in reducing lipid content is likely attributable to the cytotoxic effects of the higher concentration, as shown in the viability assays. This underscores that the beneficial metabolic effects of G1 are concentration-dependent and occur within a specific non-toxic range, a critical consideration for its potential therapeutic development. Additionally, G1 administration reduced ROS production and mildly alleviated apoptosis in HepG2 cells. Previous studies support this conclusion, demonstrating that GPER1 promotes antioxidant enzyme expression, lowers oxidative stress levels, and mitigates inflammatory damage to hepatocytes (Yao et al., 2023). *In vivo* experimental results also support this conclusion. G1 treatment improves histopathology in mouse liver tissue and hepatic lipid metabolism. Based on previous studies and the experimental results of this study, it is showed that GPER1 upregulation exerts a protective effect against hepatic steatosis.

To further explore the impact of G1 treatment, we performed untargeted LC-MS/MS-based proteomics on HepG2 cells to identify proteins upregulated by G1. We observed that GAIP-interacting protein C-terminal 1 (GIPC1) exhibited increased expression under G1 influence. GIPC1 functions as a scaffold protein supporting membrane protein trafficking (Katoh, 2013), complementing the structural characteristics of GPER1 (a seven-transmembrane receptor). Consequently, GIPC1 likely serves as either a binding partner synergizing with GPER1 or acts as a downstream molecule of GPER1. To further validate the interaction between GPER1 and GIPC1, we performed Co-IP experiments. The results confirmed our hypothesis that GPER1 and GIPC1 interact. Current research on GIPC1 primarily focuses on oncology, but its role in regulating cholesterol uptake suggests potential links to metabolic diseases (Zhang et al., 2021). This study provides the first evidence that GIPC1 may regulate hepatic lipid metabolism in MASLD by binding to GPER1. This warrants further investigation.

The enrichment analysis of KEGG and GO reveals a coherent, multi-layered response to G1 activation. The consistent and strong enrichment of the pentose phosphate pathway across both analyses highlights a fundamental rewiring of central carbon metabolism. The pentose phosphate pathway serves as the primary source of NADPH, which supports antioxidant defenses by reducing oxidized glutathione. Studies indicate that under pathological conditions where the pentose phosphate pathway is inhibited and NADPH is depleted, leading to oxidative stress, activation of GPER1 can exert cytoprotective effects. This protection is achieved by bypassing the NADPH requirement and promoting glutathione synthesis, through a mechanism independent of the classical cAMP-PKA signaling (Kilanczy et al., 2016). This suggests that the metabolic protection conferred by GPER1 may be closely linked to mitigating oxidative stress and supporting the cellular antioxidant defense system. Furthermore, the enrichment of terms related to epithelial cell migration suggests that GPER1 activation by G1 may influence hepatocyte behavior beyond metabolism, potentially affecting cell adhesion, tissue repair, or paracrine signaling within the liver microenvironment. While this finding requires future validation, it opens a new avenue for understanding the pleiotropic effects of GPER1 in liver physiology and pathology. Therefore, these findings suggest that the GPER1 agonist G1 may ameliorate MASLD through multifaceted effects. These could encompass enhanced intrinsic antioxidant defenses via metabolic rewiring, notably involving the pentose phosphate pathway, and might also extend to the modulation of reparative hepatocyte behaviors. In addition, we also performed GO and KEGG analyses on the downregulated proteome following G1 treatment in FFA-induced cells. Although the enrichment significance (FDR) did not meet the stringent cutoff of <0.05, these results offer exploratory directions for investigating G1’s mechanisms. Notably, the nicotinate and nicotinamide metabolism pathway was enriched, featuring NNMT and SIRT3, the key regulatory molecules in NAD + metabolism (Wei et al., 2023). This suggests a potential link between G1’s action and the core of cellular energy metabolism (NAD+) as well as oxidative stress regulation. It is also noteworthy that cholesterol metabolism was among the enriched pathways. While the earlier observations indicated that G1 did not significantly alter total cholesterol levels, it may influence cholesterol transport or redistribution rather than *de novo* synthesis. This warrants further investigation in future studies. Collectively, these observations point to GPER1 as a potential pleiotropic target worthy of further investigation for liver disease. Future studies are needed to validate these mechanisms and define their therapeutic relevance.

G15, a small molecule compound, is a GPER1 antagonist that is widely used (Dennis et al., 2011). It blocks signal transduction by binding to GPER1. Studies indicated that G15 may exacerbate metabolic dysfunction by inhibiting the protective functions of GPER and that it may accelerate tumor growth under specific conditions (Sharma and Prossnitz, 2021). Our findings are consistent with these observations. G15 mildly downregulated GPER1 protein expression and exacerbated steatosis in the FFA-induced steatotic cell model, characterized by significant lipid deposition and varying degrees of reduction in fatty acid oxidation-related proteins. While some fatty acid synthesis genes were upregulated. The inconsistent changes in ACC1 protein levels suggest G15 may influence fatty acid synthesis through alternative mechanisms. Additionally, we observed that G15 (1 uM) promoted ROS production in HepG2 cells and accelerated hepatocyte apoptosis. These findings indicate that G15 antagonizes the beneficial effects of GPER1 on lipid metabolism and hepatic steatosis.

This study has several limitations. Due to the difficulty in obtaining human liver samples, the statistical power of human data in this study was reduced. We will continue to collect a larger sample size in future work. Additionally, the commonly used liver cancer cell lines in the literature may not accurately represent the metabolic conditions of normal liver tissue. We will continue to refine and supplement the approach with primary liver cells or organoid models. In conclusion, this study integrates clinical liver tissue data and, for the first time, identifies the GIPC1 molecule through proteomics screening as having binding interactions with GPER1. This provides theoretical support for advancing MASLD interventions and drug development targeting the estrogen receptor GPER1.

## Data Availability

The data presented in the study are deposited in the ProteomeXchange repository, accession number PXD075389.

## References

[B1] Bardou P. Mariette J. Escudié F. Djemiel C. Klopp C. (2014). Jvenn: an interactive venn diagram viewer. BMC Bioinforma. 15 (1), 1–7. 10.1186/1471-2105-15-293 25176396 PMC4261873

[B2] Bologa C. G. Revankar C. M. Young S. M. Edwards B. S. Arterburn J. B. Kiselyov A. S. (2006). Virtual and biomolecular screening converge on a selective agonist for GPR30. Nat. Chem. Biol. 2 (4), 207–212. 10.1038/nchembio775 16520733

[B3] Cherubini A. Della Torre S. Pelusi S. Valenti L. (2024). Sexual dimorphism of metabolic dysfunction-associated steatotic liver disease. Trends Mol. Med. 30 (12), 1126–1136. 10.1016/j.molmed.2024.05.013 38890029

[B4] DeLeon C. Wang D. Q. H. Arnatt C. K. (2020). G protein-coupled estrogen receptor, GPER1, offers a novel target for the treatment of digestive diseases. Front. Endocrinol. (Lausanne) 11, 578536. 10.3389/fendo.2020.578536 33281743 PMC7689683

[B23] Della Torre S. (2026). Non-alcoholic fatty liver disease as a canonical example of metabolic inflammatory-based liver disease showing a sex-specific prevalence: relevance of estrogen signaling. Front. Endocrinology. 2020 Sept 18, 11. 10.3389/fendo.2020.572490 33071979 PMC7531579

[B5] Dennis M. K. Field A. S. Burai R. Ramesh C. Petrie W. K. Bologa C. G. (2011). Identification of a GPER/GPR30 antagonist with improved estrogen receptor counterselectivity. J. Steroid Biochem. Mol. Biol. 127 (3–5), 358–366. 10.1016/j.jsbmb.2011.07.002 21782022 PMC3220788

[B6] Dobs A. S. Nguyen T. Pace C. Roberts C. P. (2002). Differential effects of oral estrogen *versus* oral estrogen-androgen replacement therapy on body composition in postmenopausal women. J. Clin. Endocrinol. Metab. 87 (4), 1509–1516. 10.1210/jcem.87.4.8362 11932273

[B7] Estes C. Anstee Q. M. Arias-Loste M. T. Bantel H. Bellentani S. Caballeria J. (2018). Modeling NAFLD disease burden in China, France, Germany, Italy, Japan, Spain, United Kingdom, and United States for the period 2016-2030. J. Hepatol. 69 (4), 896–904. 10.1016/j.jhep.2018.05.036 29886156

[B8] Farruggio S. Cocomazzi G. Marotta P. Romito R. Surico D. Calamita G. (2020). Genistein and 17β-Estradiol protect hepatocytes from fatty degeneration by mechanisms involving mitochondria, inflammasome and kinases activation. Cell Physiol. Biochem. 54 (3), 401–416. 10.33594/000000227 32330379

[B9] Filardo E. J. Thomas P. (2012). Minireview: g protein-coupled estrogen Receptor-1, GPER-1: its mechanism of action and role in female reproductive cancer, renal and vascular physiology. Endocrinology 153 (7), 2953–2962. 10.1210/en.2012-1061 22495674 PMC3380306

[B24] Jiang S. Li H. Zhang L. Mu W. Zhang Y. Chen T. (2025). Generic diagramming platform (GDP): a comprehensive database of high-quality biomedical graphics. Nucleic Acids Research 53 (D1), D1670–D1676. 10.1093/nar/gkae973 39470721 PMC11701665

[B12] Katoh M. (2013). Functional proteomics, human genetics and cancer biology of GIPC family members. Exp. and Mol. Med. 45 (6), e26. 10.1038/emm.2013.49 23743496 PMC3701287

[B13] Kilanczyk E. Saraswat Ohri S. Whittemore S. R. Hetman M. (2016). Antioxidant protection of NADPH-depleted oligodendrocyte precursor cells is dependent on supply of reduced glutathione. ASN Neuro 8 (4), 1759091416660404. 10.1177/1759091416660404 27449129 PMC4962338

[B11] Kim J. H. Meyers M. S. Khuder S. S. Abdallah S. L. Muturi H. T. Russo L. (2014). Tissue-selective estrogen complexes with bazedoxifene prevent metabolic dysfunction in female mice. Mol. Metabolism 3 (2), 177–190. 10.1016/j.molmet.2013.12.009 24634829 PMC3953695

[B15] Lee J. H. Kim D. Kim H. J. Lee C. H. Yang J. I. Kim W. (2010). Hepatic steatosis index: a simple screening tool reflecting nonalcoholic fatty liver disease. Dig. Liver Dis. 42 (7), 503–508. 10.1016/j.dld.2009.08.002 19766548

[B14] Li L. Yao Y. Wang Y. Cao J. Jiang Z. Yang Y. (2024). G protein-coupled estrogen receptor 1 ameliorates nonalcoholic steatohepatitis through targeting AMPK-Dependent signaling. J. Biological Chemistry 300 (3), 105661. 10.1016/j.jbc.2024.105661 38246352 PMC10876613

[B16] Liu L. Zhou Y. Liu J. Zhang X. He C. Zeng X. (2025). GPER in metabolic homeostasis and disease: molecular mechanisms, nutritional regulation, and therapeutic potential. J. Transl. Med. 23 (1), 960. 10.1186/s12967-025-07005-0 40859360 PMC12382220

[B18] McKenzie J. Jaap A. J. Gallacher S. Kelly A. Crawford L. Greer I. A. (2003). Metabolic, inflammatory and haemostatic effects of a low-dose continuous combined HRT in women with type 2 diabetes: potentially safer with respect to vascular risk? Clin. Endocrinol. (Oxf) 59 (6), 682–689. 10.1046/j.1365-2265.2003.01906.x 14974908

[B20] Natale C. A. Mercado S. Zhuang R. Aguirre-Portolés C. Olayide I. Arnatt C. K. (2025). LNS8801: an enantiomerically pure agonist of the G protein-coupled estrogen receptor suitable for clinical development. Cancer Res. Commun. 5 (4), 556–568. 10.1158/2767-9764.CRC-24-0632 40066851 PMC11969138

[B21] Pham T. H. Lee G. H. Jin S. W. Lee S. Y. Han E. H. Kim N. D. (2022). Puerarin attenuates hepatic steatosis *via* G-protein-coupled estrogen receptor-mediated calcium and SIRT1 signaling pathways. Phytother. Res. 36 (9), 3601–3618. 10.1002/ptr.7526 35871535

[B22] Prossnitz E. R. Arterburn J. B. (2015). International union of basic and clinical pharmacology. XCVII. G protein–coupled estrogen receptor and its pharmacologic modulators. Pharmacol. Rev. 67 (3), 505–540. 10.1124/pr.114.009712 26023144 PMC4485017

[B17] Ricchi M. Odoardi MR. Carulli L. Anzivino C. Ballestri S. Pinetti A. (2009). Differential effect of oleic and palmitic acid on lipid accumulation and apoptosis in cultured hepatocytes. J. Gastroenterology Hepatology 24 (5), 830–840. 10.1111/j.1440-1746.2008.05733.x 19207680

[B19] Rinella M. E. Lazarus J. V. Ratziu V. Francque S. M. Sanyal A. J. Kanwal F. (2023). A multisociety Delphi consensus statement on new fatty liver disease nomenclature. Hepatol. Baltim. Md 78 (6), 1966–1986. 10.1097/HEP.0000000000000520 37363821 PMC10653297

[B25] Sharma G. Prossnitz E. R. (2021). Targeting the G protein-coupled estrogen receptor (GPER) in obesity and diabetes. Endocr. Metab. Sci. 2, 100080. 10.1016/j.endmts.2021.100080 35321004 PMC8936744

[B10] Sharma G. Hu C. Staquicin D. I. Brigman J. L. Liu M. Mauvais-Jarvis F. (2020). Preclinical efficacy of the GPER-Selective agonist G-1 in mouse models of obesity and diabetes. Sci. Translational Medicine 12 (528). 10.1126/scitranslmed.aau5956 PMC7083206 31996464

[B26] Song Y. Guo W. Li Z. Guo D. Li Z. Li Y. (2022). Systemic immune-inflammation index is associated with hepatic steatosis: evidence from NHANES 2015-2018. Front. Immunol. 13, 1058779. 10.3389/fimmu.2022.1058779 36466832 PMC9718528

[B27] Wang Y. Chen C. Chen J. Sang T. Peng H. Lin X. (2022). Overexpression of NAG-1/GDF15 prevents hepatic steatosis through inhibiting oxidative stress-mediated dsDNA release and AIM2 inflammasome activation. Redox Biol. 52, 102322. 10.1016/j.redox.2022.102322 35504134 PMC9079118

[B28] Wei X. Wei C. Tan Y. Dong X. Yang Z. Yan J. (2023). Both prolonged high-fat diet consumption and calorie restriction boost hepatic NAD+ metabolism in mice. J. Nutr. Biochem. 115, 109296. 10.1016/j.jnutbio.2023.109296 36849030

[B29] Yao Y. Wang H. Yang Y. Jiang Z. Ma H. (2023). Dehydroepiandrosterone protects against oleic acid-triggered mitochondrial dysfunction to relieve oxidative stress and inflammation *via* activation of the AMPK-Nrf2 axis by targeting GPR30 in hepatocytes. Mol. Immunol. 155, 110–123. 10.1016/j.molimm.2023.01.008 36773597

[B30] Zhang Z. Zhou Q. Liu R. Liu L. Shen W. J. Azhar S. (2021). The adaptor protein GIPC1 stabilizes the scavenger receptor SR-B1 and increases its cholesterol uptake. J. Biol. Chem. 296, 100616. 10.1016/j.jbc.2021.100616 33811857 PMC8093464

