# Supplementary material for: GPER1 as a therapeutic target in MASLD: evidence for steatosis attenuation by agonist G1 in preclinical models
Source: Front Pharmacol. 2026 Mar 18;17:1764287. doi: 10.3389/fphar.2026.1764287 (PMC13038608; doi:10.3389/fphar.2026.1764287)
Supplement: Supplementary file 2 [file Table1.docx]

**Table S1 The antibody information used in this article.**

| **Antibodies** | **Application** | **Dilution** | **Source** | **Identifier** |
| --- | --- | --- | --- | --- |
| Rabbit monoclonal anti-beta actin | WB | 1:1000 | Servicebio | Cat#GB15003-100 |
| Rabbit polyclonal anti-GPER1 | WB | 1:1000 | Novus Biologicals | Cat#NBP1-31239 |
| Rabbit polyclonal anti-GPER1 | IHC | 1:100 | Abcam | Cat#ab39742 |
| Rabbit polyclonal anti-SREBP1c | WB | 1:1000 | Proteintech | Cat#14088-1-AP |
| Mouse monoclonal anti-ACC1 | WB | 1:10000 | Proteintech | Cat#67373-1-Ig |
| Rabbit polyclonal anti-CPT1A | WB | 1:5000 | Proteintech | Cat#15184-1-AP |
| Mouse monoclonal anti-PGC1a | WB | 1:5000 | Proteintech | Cat#66369-1-Ig |
| Rabbit polyclonal anti-ACOX1 | WB | 1:1000 | Proteintech | Cat#10957-1-AP |
| Mouse monoclonal anti-PPAR alpha | WB | 1:1000 | Proteintech | Cat#66826-1-Ig |
| Rabbit monoclonal anti-ATGL | WB | 1:1000 | HUABIO | Cat#HA721951 |
| Rabbit monoclonal anti-phospho-ACC1 (Ser79) | WB | 1:1000 | Cell Signaling Technology | Cat#11818 |
| Anti-rabbit IgG (H+L) (DyLight 680 Conjugate) | WB | 1:15000 | Cell Signaling Technology | Cat#5366 |
| Anti-mouse IgG (H+L) (DyLight 680 Conjugate) | WB | 1:15000 | Cell Signaling Technology | Cat#5470 |
| Anti-rabbit IgG (H+L) (DyLight 800 4X PEG Conjugate) | WB | 1:30000 | Cell Signaling Technology | Cat#5151 |
| HRP conjugated goat anti-rabbit IgG (H+L) | IHC | 1:200 | Servicebio | Cat#GB23303 |
