# Supplementary material for: GPER1 as a therapeutic target in MASLD: evidence for steatosis attenuation by agonist G1 in preclinical models
Source: Front Pharmacol. 2026 Mar 18;17:1764287. doi: 10.3389/fphar.2026.1764287 (PMC13038608; doi:10.3389/fphar.2026.1764287)
Supplement: Supplementary file 3 [file Table2.docx]

**Table S2. Primer sequences used for Real-Time Quantitative PCR.**

| Gene | Primer | Sequence (5’-3’) |
| --- | --- | --- |
| β-Actin | Forward | TCACCAACTGGGACGACATG |
|  | Reverse | GAGGCGTACAGGGATAGCAC |
| GPER1 | Forward | TTCCGCGAGAAGATGACCATCC |
|  | Reverse | TAGTACCGCTCGTGCAGGTTGA |
| SREBP1c | Forward | ACTTCTGGAGGCATCGCAAGCA |
|  | Reverse | AGGTTCCAGAGGAGGCTACAAG |
| FASN | Forward | TTCTACGGCTCCACGCTCTTCC |
|  | Reverse | GAAGAGTCTTCGTCAGCCAGGA |
| SCD1 | Forward | CCTGGTTTCACTTGGAGCTGTG |
|  | Reverse | TGTGGTGAAGTTGATGTGCCAGC |
| ACC1 | Forward | TTCACTCCACCTTGTCAGCGGA |
|  | Reverse | GTCAGAGAAGCAGCCCATCACT |
| CD36 | Forward | CAGGTCAACCTATTGGTCAAGCC |
|  | Reverse | GCCTTCTCATCACCAATGGTCC |
| PPARγ | Forward | AGCCTGCGAAAGCCTTTTGGTG |
|  | Reverse | GGCTTCACATTCAGCAAACCTGG |
| HMGCR | Forward | GACGTGAACCTATGCTGGTCAG |
|  | Reverse | GGTATCTGTTTCAGCCACTAAGG |
| SREBP2 | Forward | CTCCATTGACTCTGAGCCAGGA |
|  | Reverse | GAATCCGTGAGCGGTCTACCAT |
| LDLR | Forward | GGTCCAGTAGATGTTGCTGTGG |
|  | Reverse | GAATCTACTGGTCTGACCTGTCC |
| LRP1 | Forward | CAACGGCATCTCAGTGGACTAC |
|  | Reverse | TGTTGCTGGACAGAACCACCTC |
