# Supplementary figures and images for: GPER1 as a therapeutic target in MASLD: evidence for steatosis attenuation by agonist G1 in preclinical models
Source: Front Pharmacol. 2026 Mar 18;17:1764287. doi: 10.3389/fphar.2026.1764287 (PMC13038608; doi:10.3389/fphar.2026.1764287)

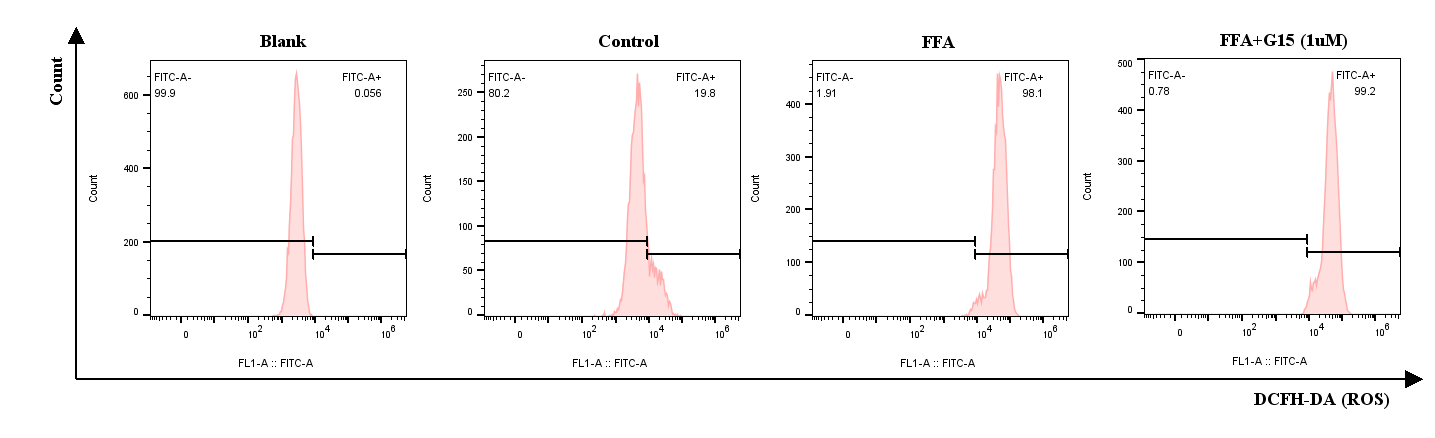

Supplement: Supplementary file 5 [file Supplementaryfile1.zip › Figure S15.tiff]

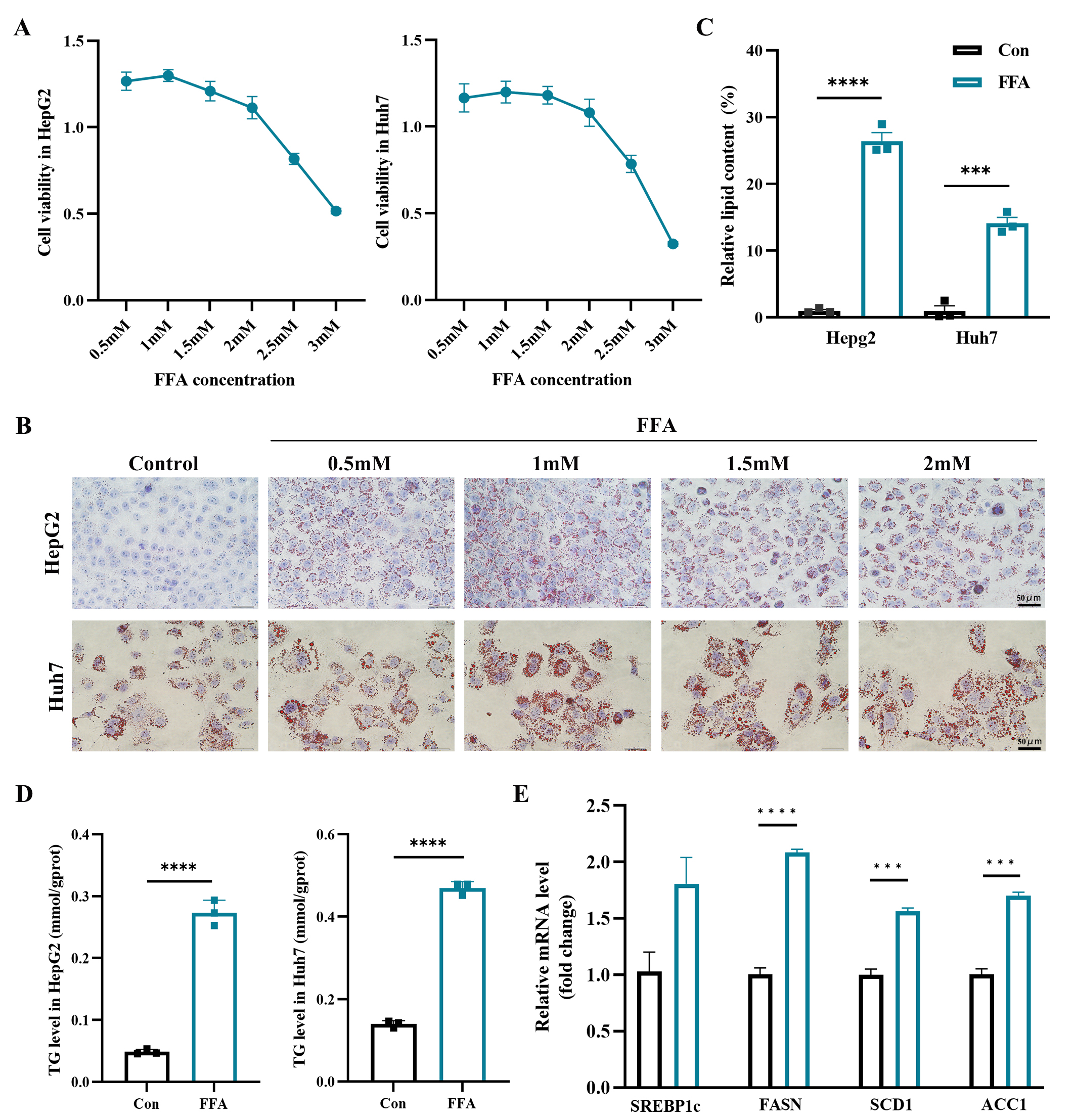

Supplement: Supplementary file 5 [file Supplementaryfile1.zip › Figure S1.jpg]

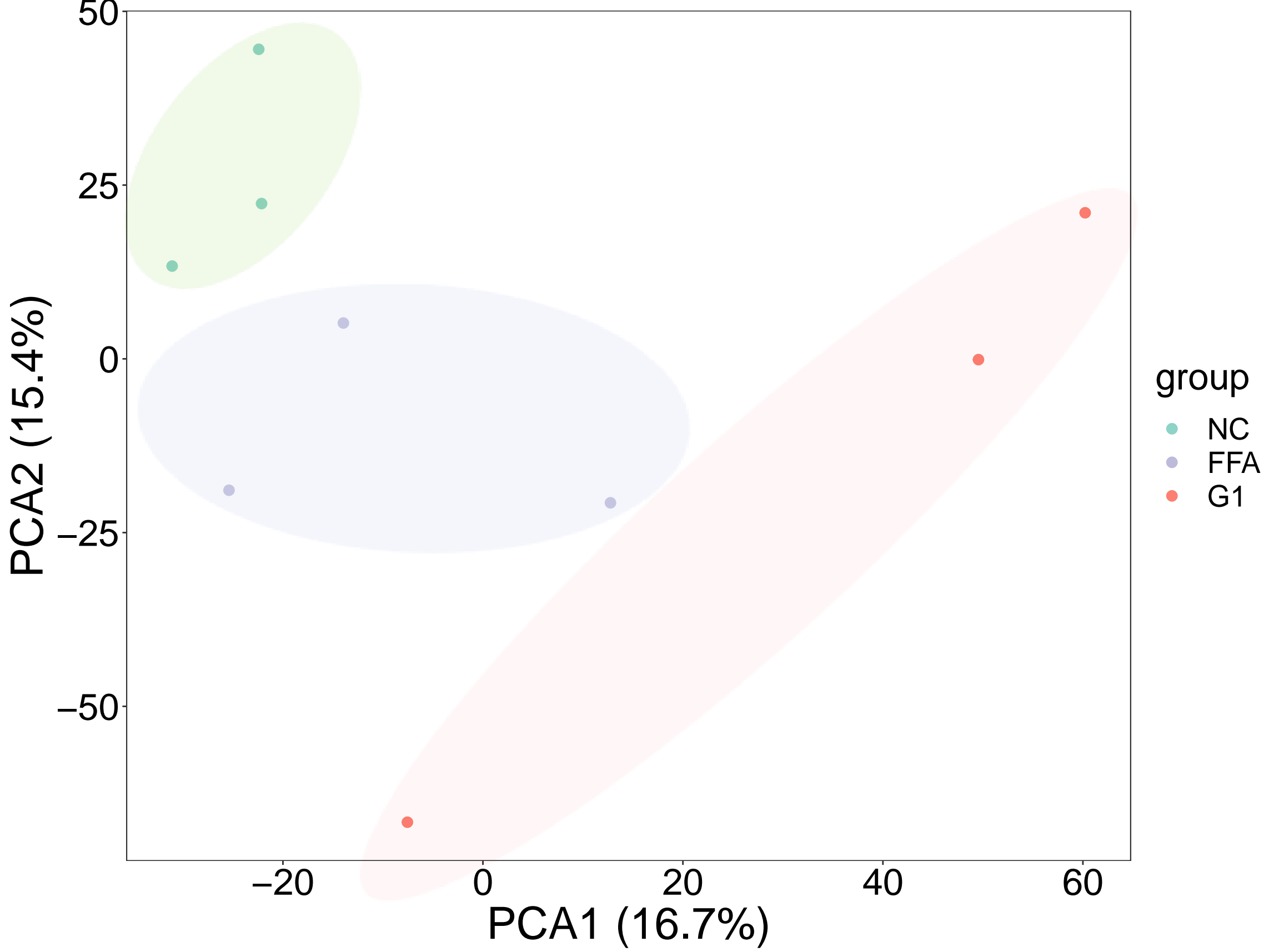

Supplement: Supplementary file 5 [file Supplementaryfile1.zip › Figure S2.pdf]

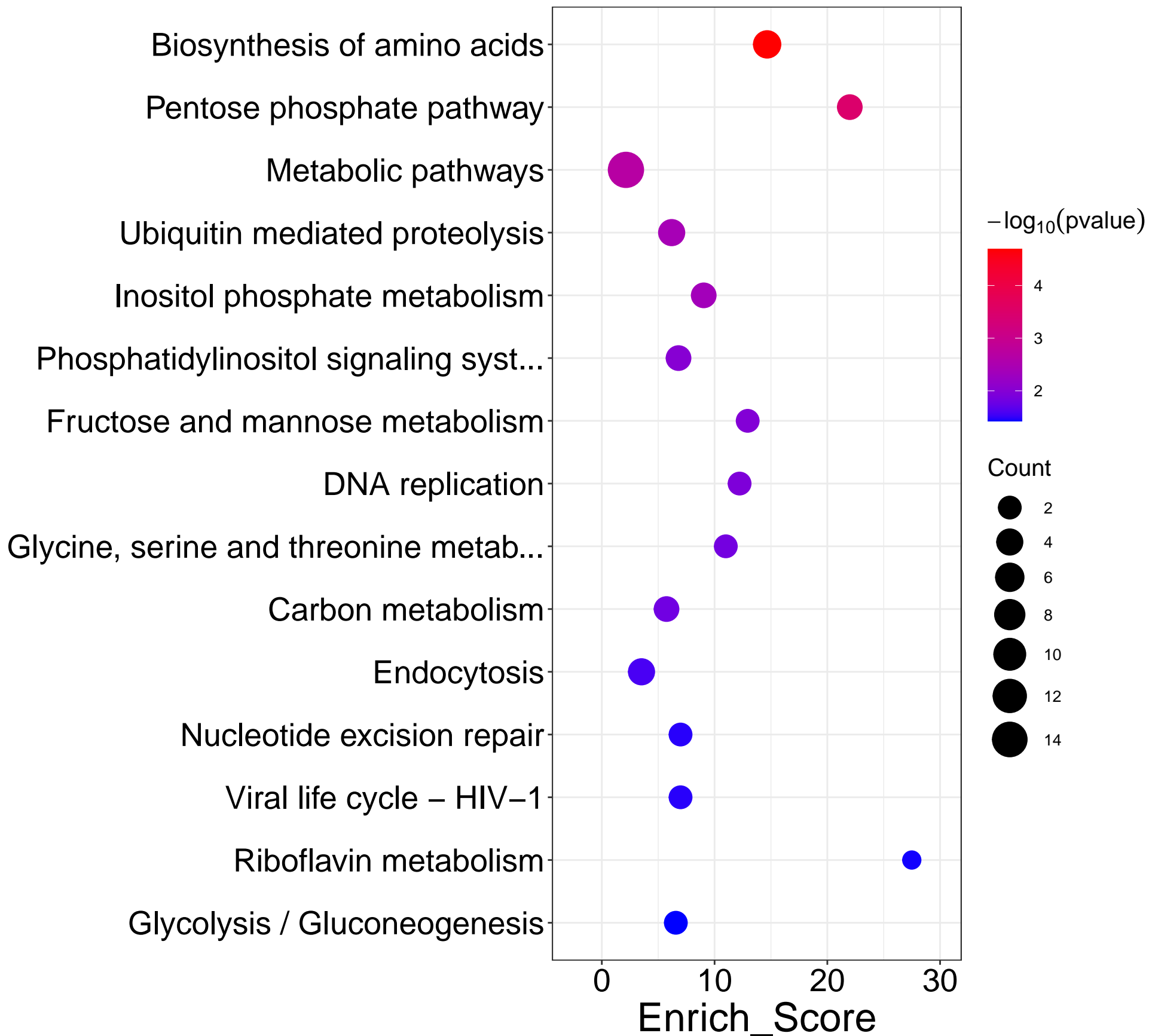

Supplement: Supplementary file 5 [file Supplementaryfile1.zip › Figure S3.pdf]

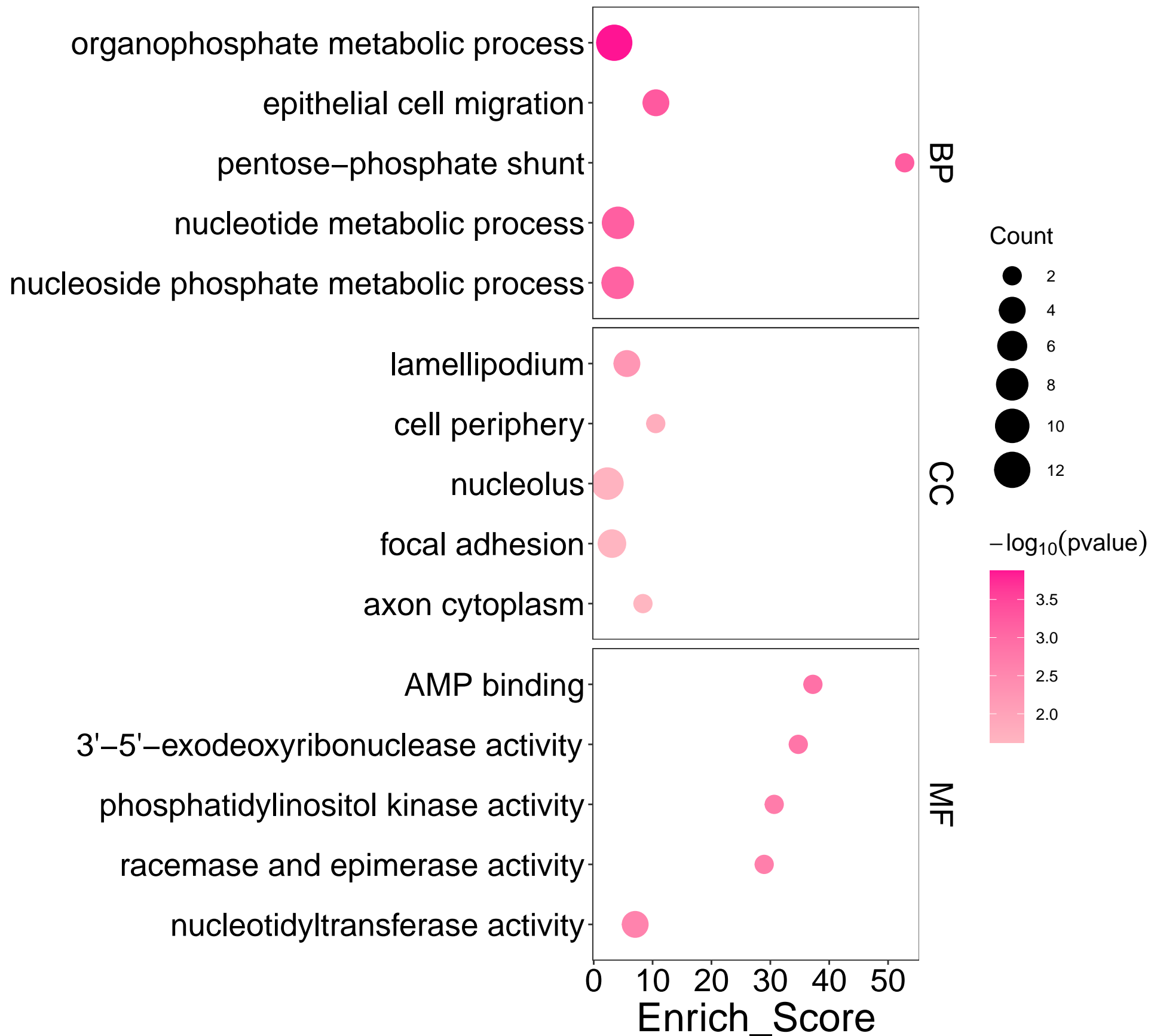

Supplement: Supplementary file 5 [file Supplementaryfile1.zip › Figure S4.pdf]

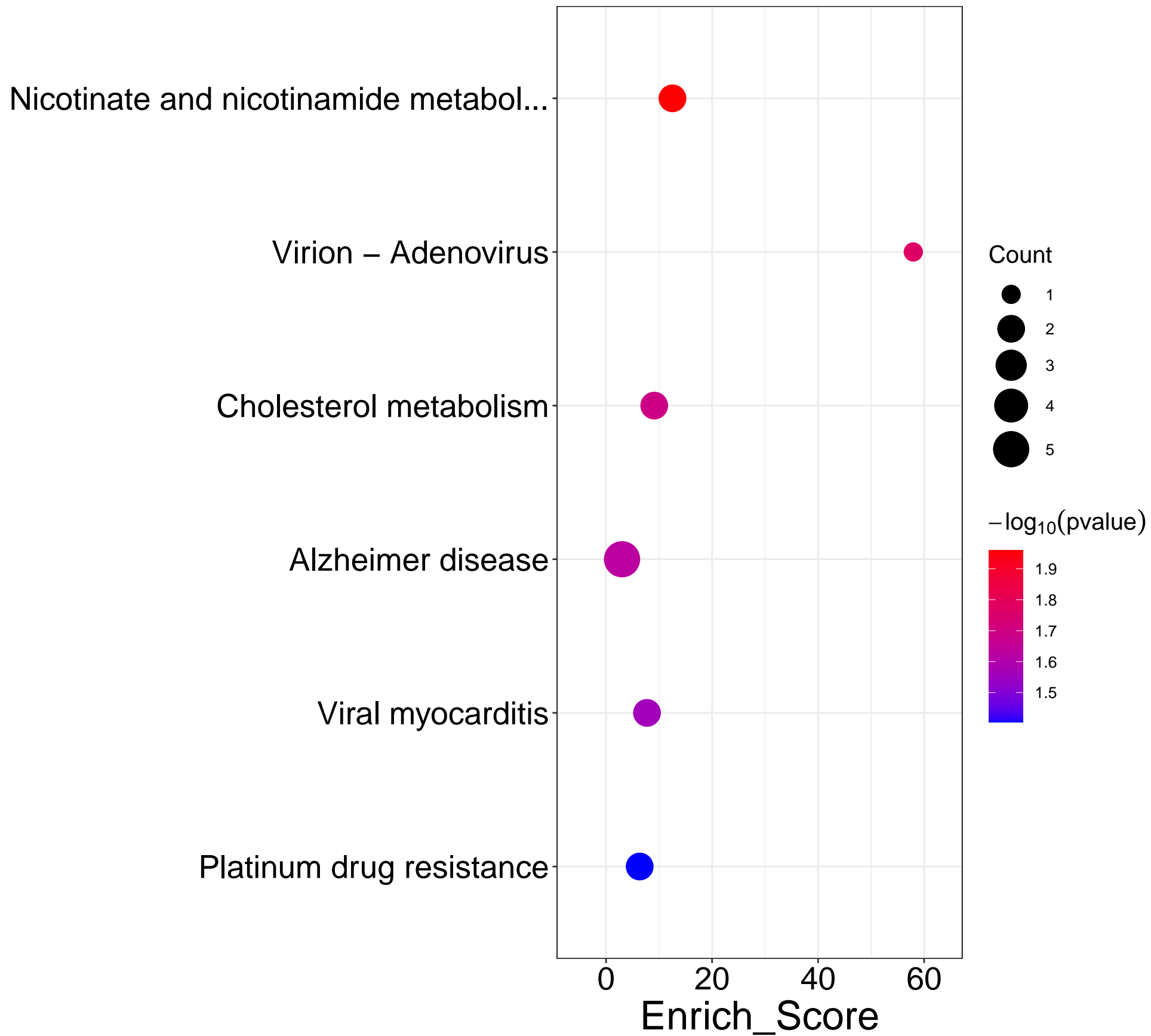

Supplement: Supplementary file 5 [file Supplementaryfile1.zip › Figure S5.pdf]

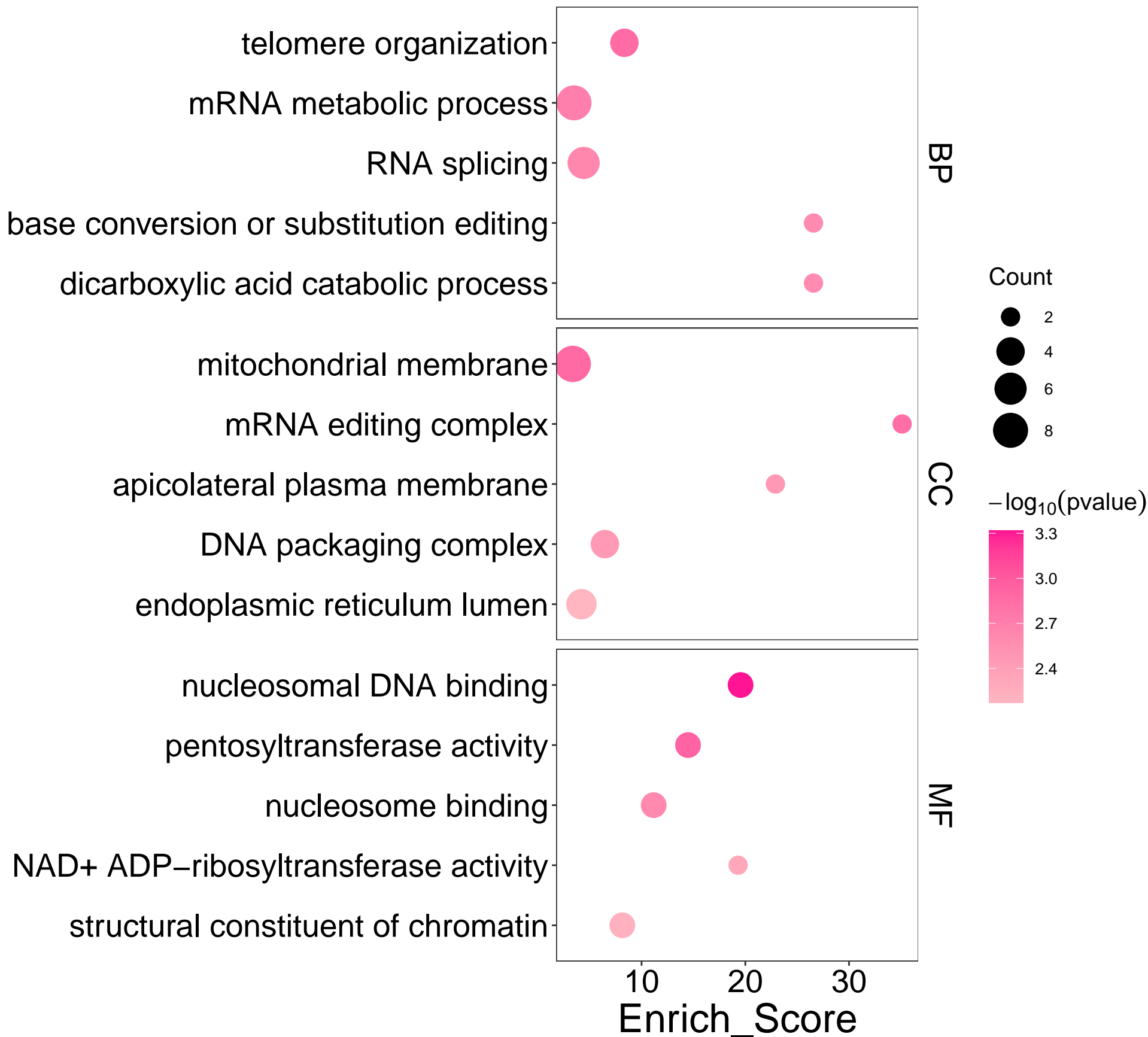

Supplement: Supplementary file 5 [file Supplementaryfile1.zip › Figure S6.pdf]

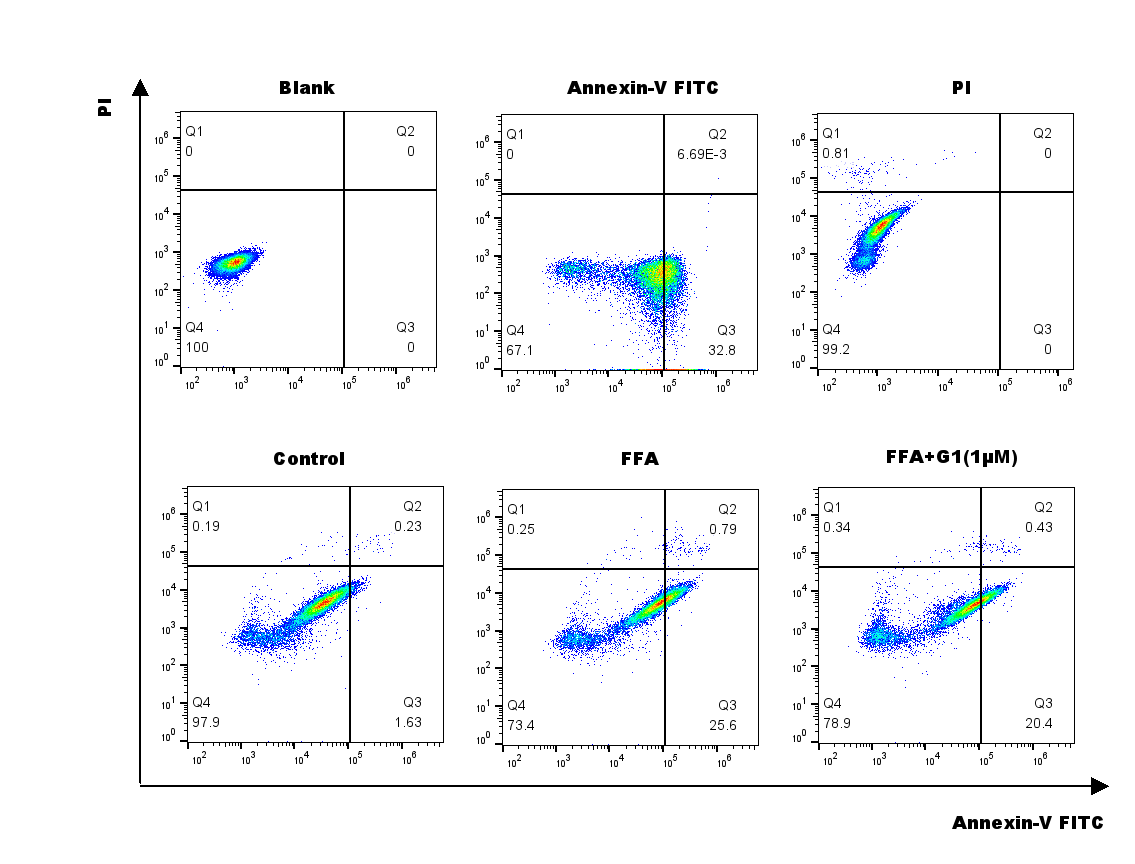

Supplement: Supplementary file 5 [file Supplementaryfile1.zip › Figure S7.tiff]

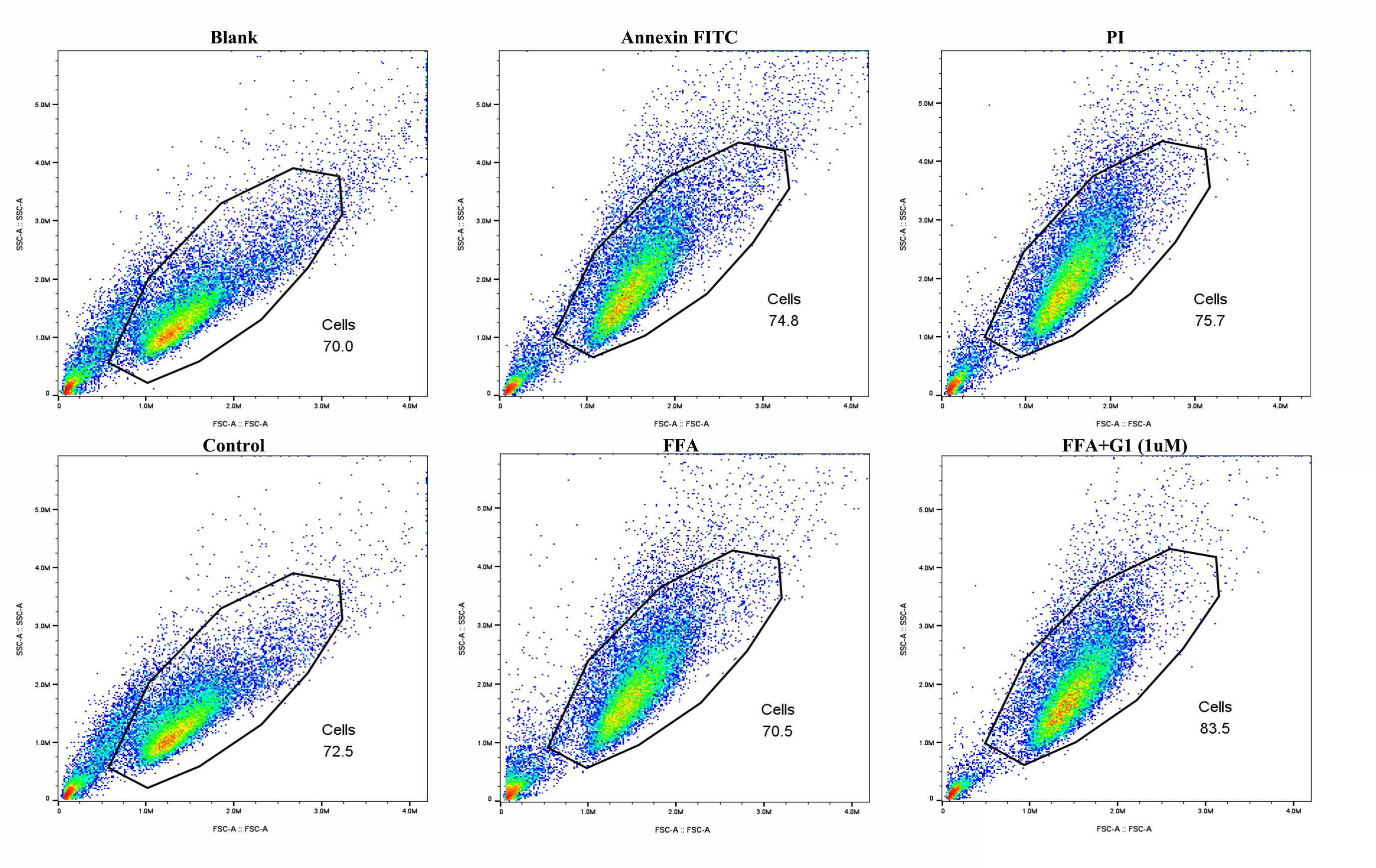

Supplement: Supplementary file 5 [file Supplementaryfile1.zip › Figure S8.jpg]

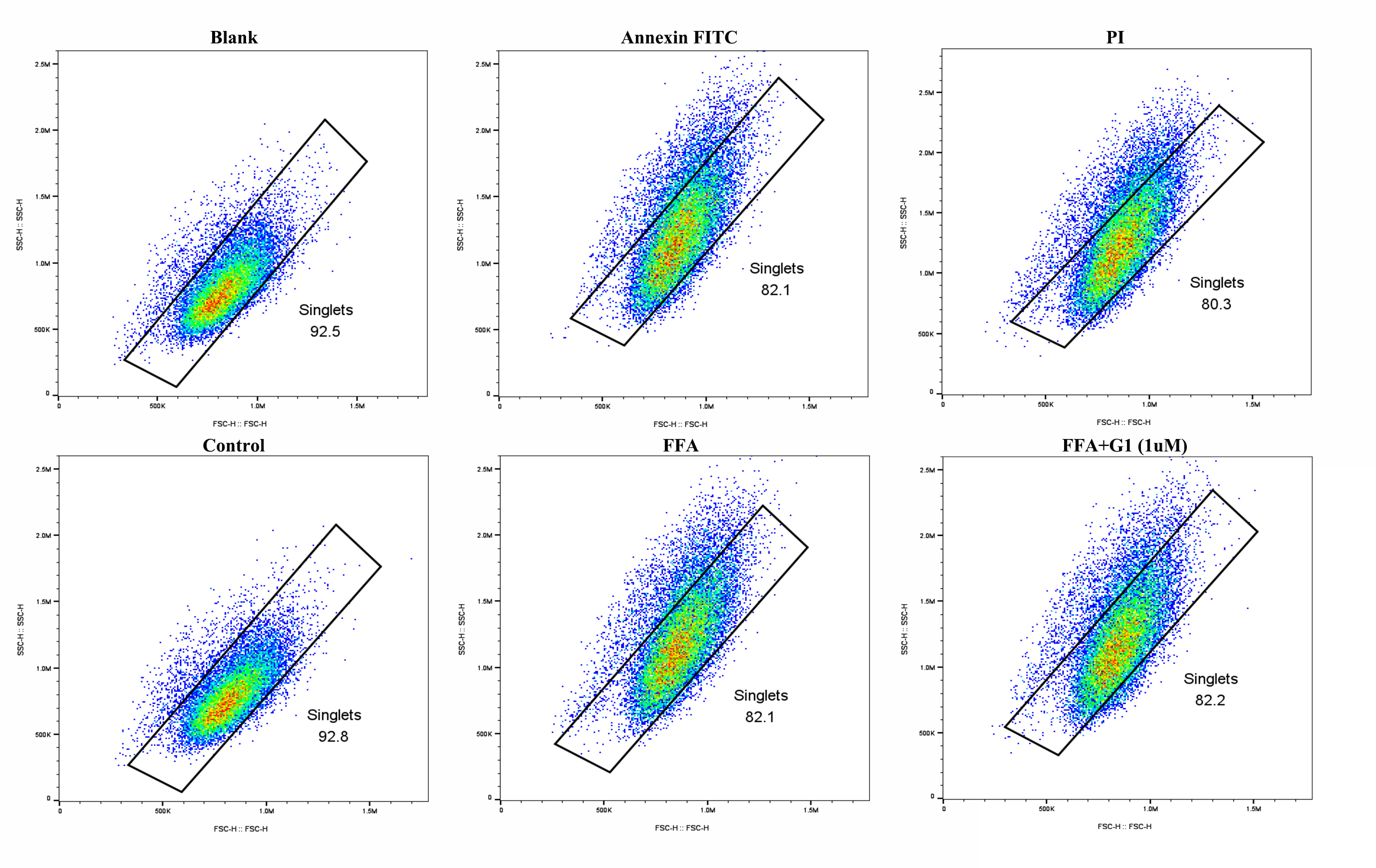

Supplement: Supplementary file 5 [file Supplementaryfile1.zip › Figure S9.jpg]

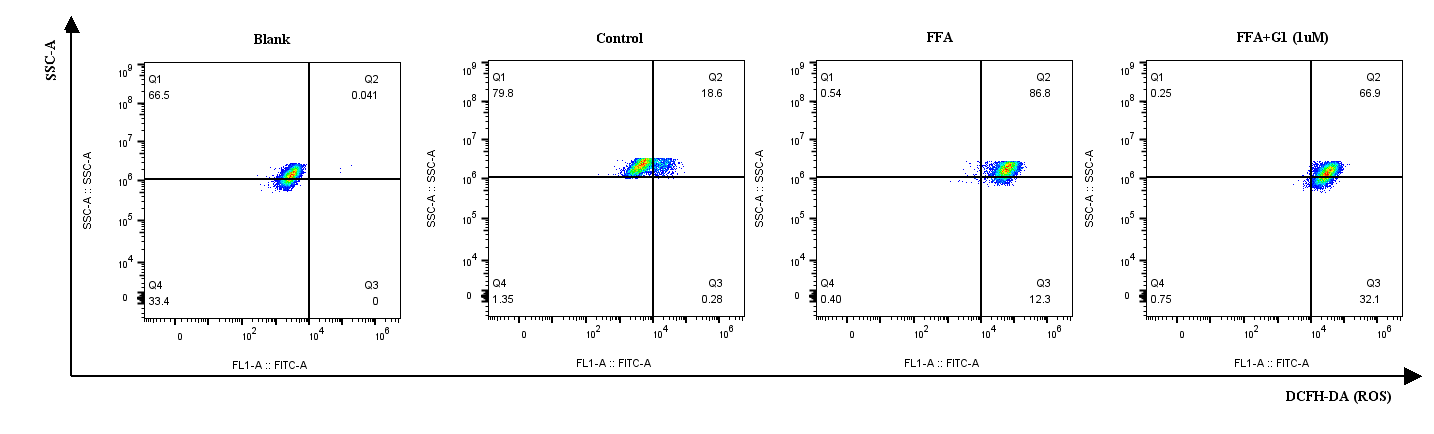

Supplement: Supplementary file 5 [file Supplementaryfile1.zip › Figure S10.tiff]

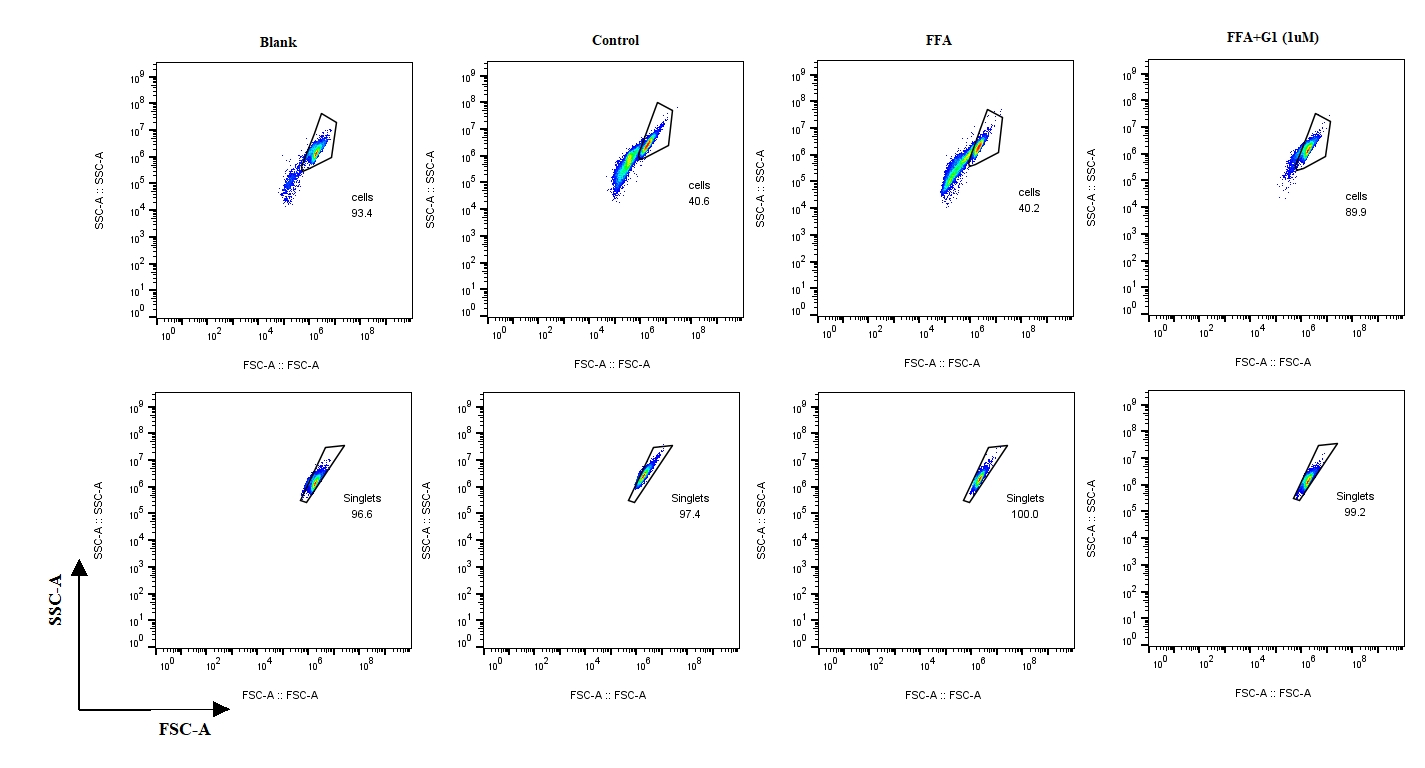

Supplement: Supplementary file 5 [file Supplementaryfile1.zip › Figure S11.jpg]

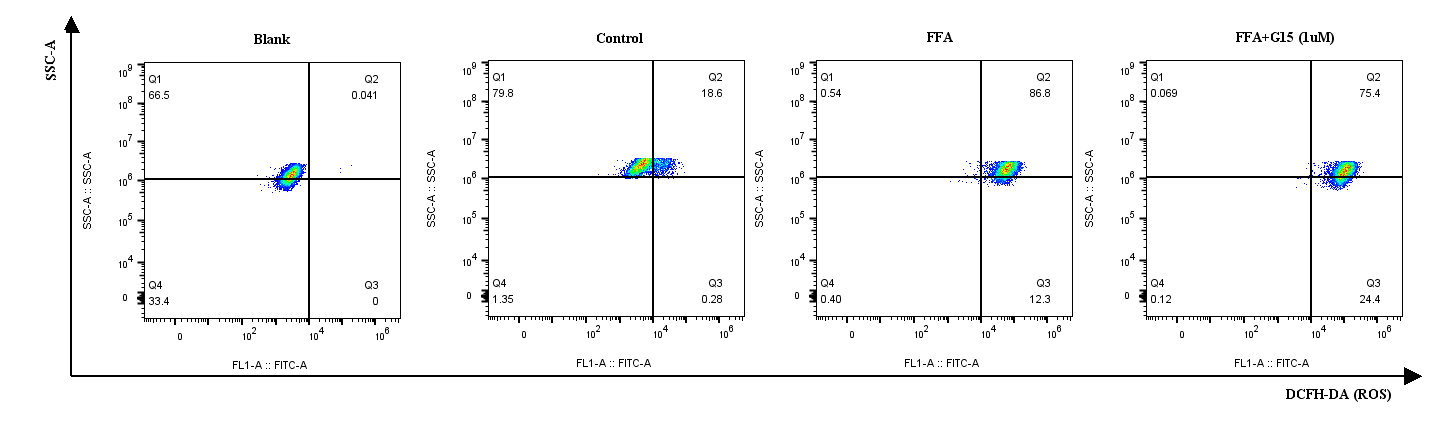

Supplement: Supplementary file 5 [file Supplementaryfile1.zip › Figure S12.tiff]

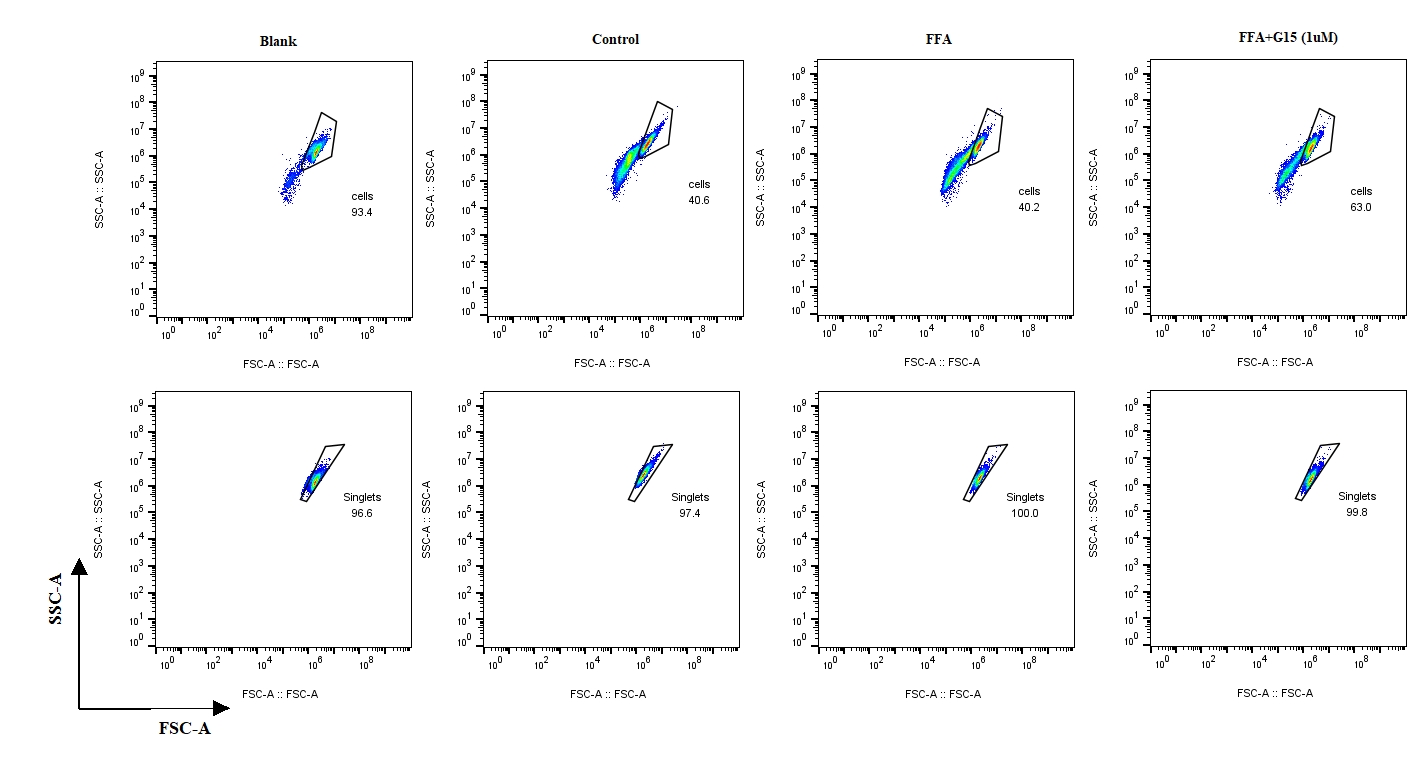

Supplement: Supplementary file 5 [file Supplementaryfile1.zip › Figure S13.jpg]

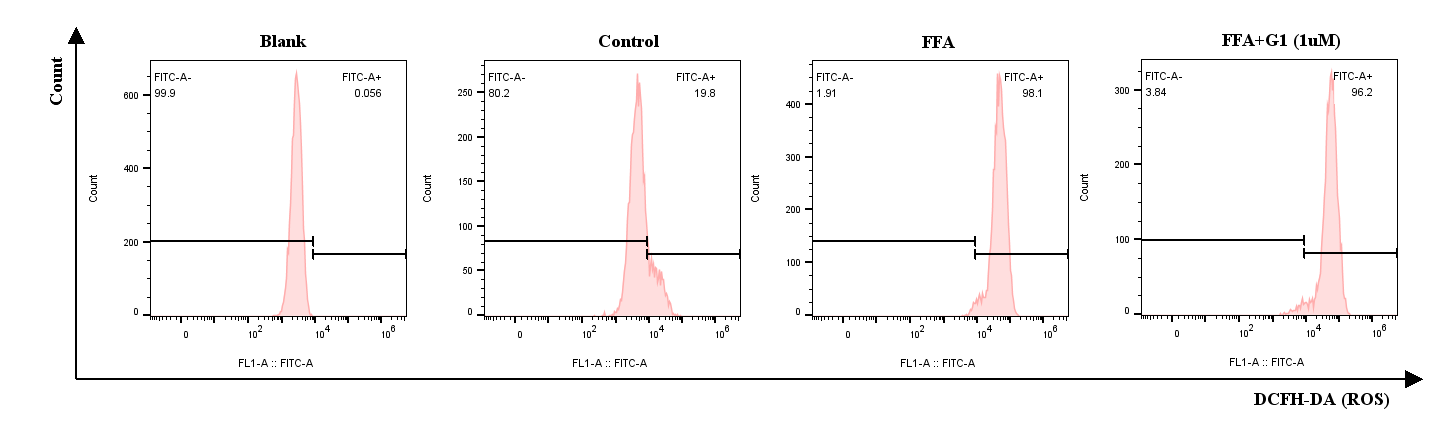

Supplement: Supplementary file 5 [file Supplementaryfile1.zip › Figure S14.tiff]
